# Deciphering the soil–rice continuum: transfer dynamics and dietary risks of potentially toxic elements in paddy fields near a coal-fired thermal power plant

**DOI:** 10.1039/d6ra06383g

**Published:** 2026-09-09

**Authors:** Likhitha Chekuri, Zahid Bashir, Deep Raj

**Affiliations:** a Department of Environmental Science and Engineering, School of Engineering and Sciences, SRM University-AP Amaravati Andhra Pradesh 522240 India likhitha_chekuri@srmap.edu.in zahidbashir5175@gmail.com deepraj2587@gmail.com

## Abstract

Thermal power plants (TPPs) located near intensively cultivated rice-growing landscapes act as significant sources of potentially toxic element (PTE) contamination. This study explored the distribution, soil-to-rice transfer, and dietary health risk of PTEs through paddy ingestion in agricultural soil around a coal-based TPP. The contamination status of soil, uptake of PTEs in plants, and dietary exposure risk were assessed using soil and plant samples from 47 paddy cultivation locations within a 10 km buffer zone radius. The results indicated the highest enrichment of Cd (contamination factor (CF) ≈ 7.5), followed by substantial enrichment of nickel (Ni), with several sites exceeding the extremely high pollution threshold and Ni concentrations exceeding the soil guideline value for international standards. Moderate to considerable contamination of soil by PTEs was indicated overall, with an average pollution load index (PLI) ≈ 1.48. Grain contamination was also evident, with 72% of sites exceeding the permissible limit for Cr and 66% for both Pb and Ni. Bioaccumulation and translocation factors revealed that As, Pb, and Cr were preferentially retained in roots, whereas Cd and Ni showed comparatively greater internal mobility. The health risk assessment based on rice consumption revealed elevated non-carcinogenic risk for children (HI = 13.5), predominantly driven by As, and high carcinogenic risk for adults (TCR = 1.72 × 10^−2^), with As, Cd, and Ni identified as the major contributors. Sensitivity analysis indicated that rice ingestion rate and exposure frequency were the principal contributors to risk variability, exceeding the influence of PTE concentration in rice grains. These findings demonstrate that dietary risk may remain substantial despite moderate composite soil pollution and highlight the need for integrated soil–plant–grain monitoring around coal-fired thermal power plants.

## Abstract 2

Thermal power plants (TPPs) located near intensively cultivated rice-growing landscapes act as significant sources of potentially toxic element (PTE) contamination.

## Graphical abstract

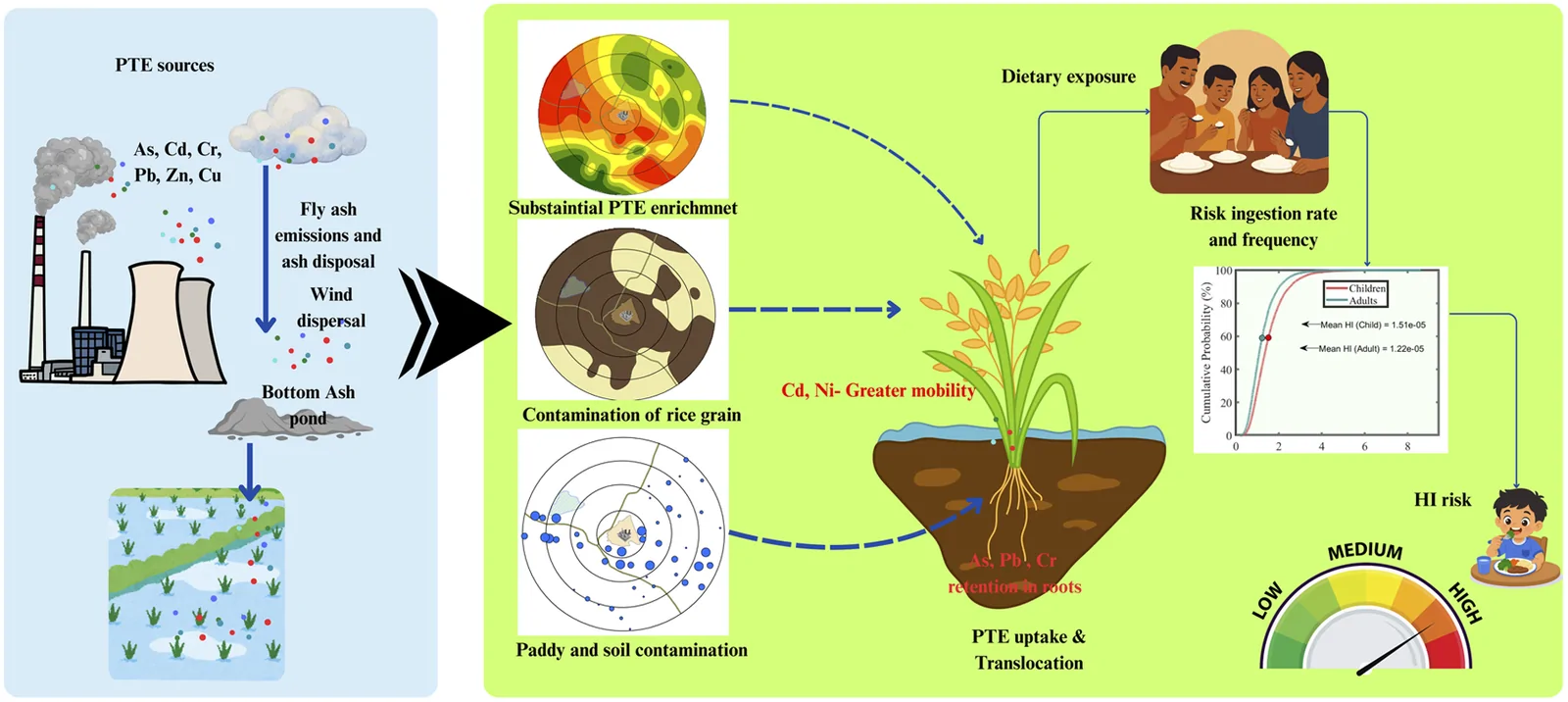

## 1 Introduction

Potentially toxic elements (PTEs), including arsenic (As), cadmium (Cd), chromium (Cr), lead (Pb), and nickel (Ni), are known persistent and non-biodegradable environmental contaminants.^1,2^ The accumulation of PTEs fundamentally degrades soil matrix quality and suppresses agricultural fertility.^3^ In addition, the accumulation of contaminants in agricultural food products can be harmful to food security as well as human health.^4^ Coal-fired thermal power plants (TPPs) are important point sources of PTE emissions and their associated spatially isolated pollution sources. Coal burning, stack emissions, fly ash disposal and wastewater treatment continuously release As, Cd, Cr and Pb into the surrounding environment resulting in the creation of pollution gradients and adjacent agricultural lands become long-term sinks for PTEs.^5–7^ In addition to industrial point sources, PTEs in agricultural systems can also originate from mining and ore-processing activity, agricultural residue burning, vehicular traffic emissions, and the application of phosphate fertilisers and pesticides.^8,9^ The most critical situation of PTE pollution occurs in an area where agricultural land around the TPP grows staple food crops, such as rice (*Oryza sativa* L.).^10^ Rice plants have a high capacity to uptake toxic metals from contaminated soil and water sources for their growth and development.^11,12^

Rice is the major food item consumed by the Asian population. Consequently, rice may also be a major source of dietary exposure to PTEs in humans. Rice consumption is often linked with various carcinogenicity, neurotoxicity, as well as renal, liver and heart diseases, due to its high accumulation of toxic metals.^13,14^ This emphasizes the importance of estimating PTEs transfer from soil into rice grains for human health risk assessments.

The transfer of PTEs from soil to plant is governed by complex and highly site-specific soil–plant interactions.^15^ Critical soil properties including pH, organic matter content, cation exchange capacity, and texture strongly regulate metal speciation, solubility, and phytoavailability.^16–19^ These processes are quantitatively analysed using bioaccumulation and translocation efficiency, which define the efficiency and directionality of metal movement within the plant system.^20^ Consequently, a robust risk assessment necessitates the integration of translocation metrics with fundamental soil physicochemical controls.

Despite extensive evidence of metal contamination in agricultural soils near industrial sources, key gaps remain in understanding compartment-specific metal transfer and dietary risk within the soil–rice–human pathway. Most studies use a single soil-to-grain transfer factor, overlooking metal uptake and translocation among roots, shoots, and grains and limiting identification of specific accumulation or attenuation points. Moreover, the relative contributions of exposure behaviour and grain metal concentrations to probabilistic dietary risk remain poorly characterized. Addressing these gaps is crucial for better understanding metal transfer in the soil–rice system and accurately assessing dietary risks in contaminated agricultural environments.

The present study therefore examined whether spatial proximity and directional alignment with the TPP and ash pond influence the intensity of PTE enrichment in surrounding soils. Such enrichment may subsequently affect PTE bioaccumulation, translocation to rice grains, and dietary health risks, particularly among children due to their lower body weight and relatively higher rice ingestion rate. The specific objectives were aimed to: (1) map the spatial variability, contamination intensity, and PTE induced ecological risk in paddy soils in the vicinity of the TPP; (2) evaluate PTEs bioconcentration and internal translocation in rice plants; (3) determine the influence of soil physicochemical properties on metal translocation; (4) assess the safety of rice grains and predict the non-carcinogenic (HI) and carcinogenic (CR) risk due to the consumption. This study presents a comprehensive tool for assessing the impact of point-source industries that has practical relevance for the safety of agricultural produce and its governance.

## 2 Materials and methods

### 2.1 Study area description

**Fig. 1:**
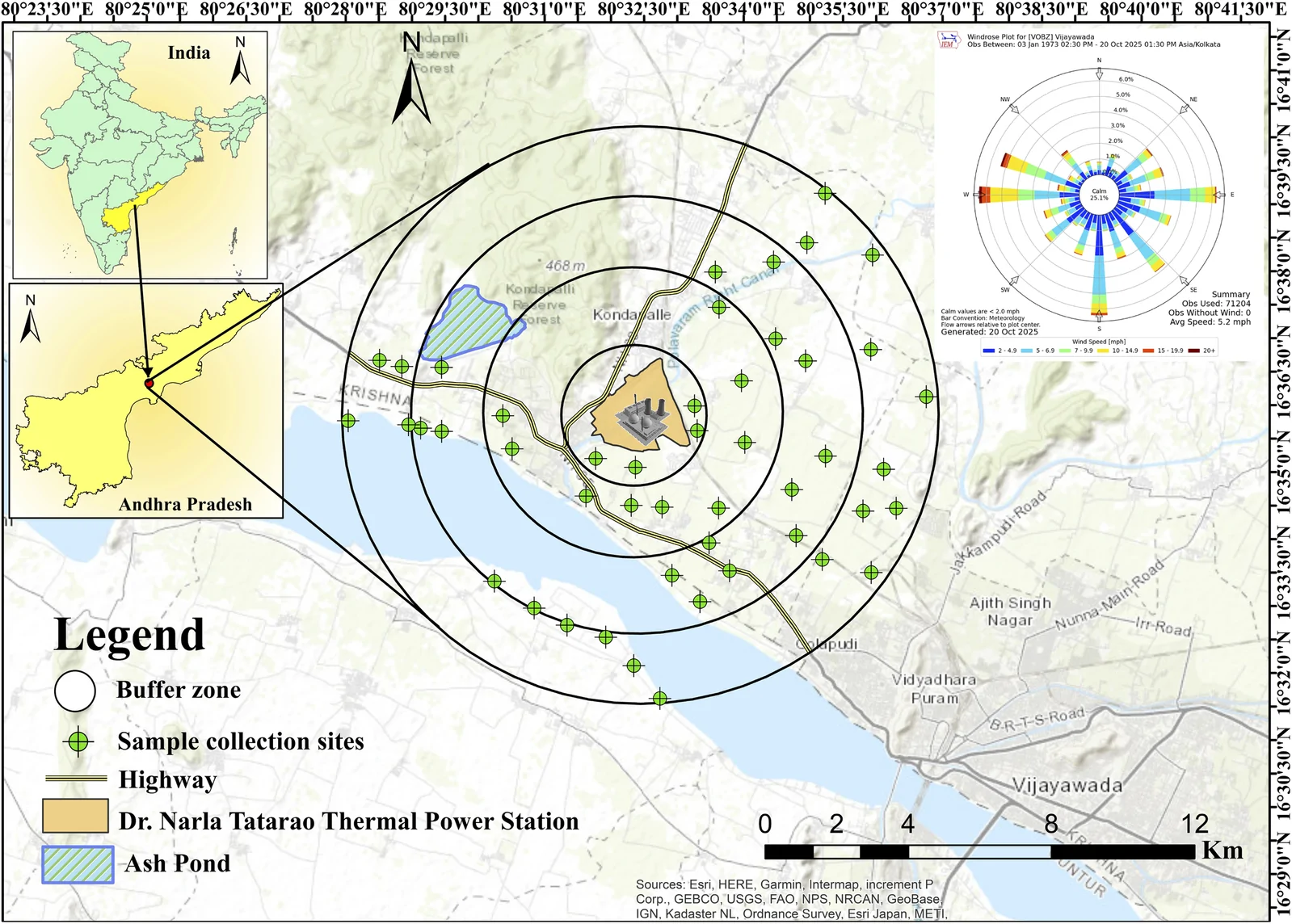
Study area map showing thermal power plant, associated ash pond, buffer zones, and sampling sites.

The current research was conducted in paddy fields around Dr NTTPS, a coal-based thermal power station, located in Andhra Pradesh, India, to assess its potential influence on the surrounding agricultural environment (Fig. 1). The TPP is located near the left bank of Krishna River in a predominantly agricultural and paddy-dominated landscape (16°35′33″ N and 80°32′03″ E). Two of the most popular rice varieties grown in the area are Samba Mahsuri (Bapatla Samba Mahsuri; BPT 5204), and MTU 1010 (Maruteru 1010, Cottondora Sannalu) which constitute the major rice varieties cultivated in the study area.^21^

The Dr NTTPS is a coal-based thermal power plant with an installed capacity of 1760 MW, consisting of several units commissioned between 1979 to 2009 and an 800 MW supercritical expansion unit (Stage-V) proposed for construction near the existing facility. The TTP uses coal as the only fuel in the proposed supercritical unit and has been designed to burn coal with approximately 38% ash content. Continuous coal combustion and material handling generates approximately ∼4560 t per day of ash with ∼3648 t per day fly ash and ∼912 t per day bottom ash, constituting a major and persistent source of ash residues potentially enriched with PTEs. Hence, ash handling is one of the dominant land-use patterns in the power plant site with ∼230 acres allocated for ash pond expansion and ∼85 acres for the main plant and its associated infrastructure. Fly ash is primarily managed through dry disposal in the ash pond.^22^

The predominant wind direction is southwest during the monsoon and northeast during the non-monsoon period. As per the 2012 EIA terms of reference, the environmental impact zone of the plant extends to a radial buffer of 8–10 km covering several residential villages and agricultural areas.^23^ Agriculture in the impact zone is mostly dependent on irrigation from the Krishna River system, thereby increasing the chances of contaminant transfer between different environmental compartments. The region experiences high temperatures in the summer season and an annual mean rainfall of 973 mm. The predominant soil types are clay to gravelly clayey moderately deep dark brown soils that support high-intensity paddy cultivation. The proximity of extensively cultivated paddy fields to the TPP provides an appropriate setting for investigating PTE contamination, transfer within the soil–rice system, and potential implications for soil and food quality.

### 2.2 Sample collection and processing

To characterize the spatial gradient of contamination originating from the thermal power plant, a distance-stratified random sampling approach was applied. The 10 km study area (EIA ToR, 2012) was split into four concentric buffer zones (0–2, 2–4, 4–6, and 6–10 km). Using a grid-overlay, 47 sampling locations were randomly positioned within each zone, restricted to operational rice fields where the target cultivar is grown. Soil and rice plant samples were collected simultaneously during the November 2025 harvest from each selected location. For soil sampling (0–15 cm depth), a stainless-steel auger was used to collect the rhizospheric soil associated with the rice plants. For each site, three soil cores (500 g each) and three rice plants (including roots, shoots and grains) were sampled within a 2 m radius from the point of the soil sampling. Soil cores were composited and homogenized by the quartering method yielding one final composite sample. Likewise, the three rice plants were composited to yield a single composite plant sample. At each site, three replicates for both plant and soil samples were composited and treated as a single unit. Cultivar-specific distinctions were not made during sampling, and the collected rice grain samples were therefore not differentiated according to cultivar type (Samba Mahsuri or MTU 1010) at the individual sampling-site level. Soil samples were stored in food-grade polyethylene bags whereas plant samples were placed in paper bags and shipped to the lab within 6 h of collection. Background soil samples (*n* = 3) were collected from agricultural fields located approximately 20 km upwind (northeast) of the TPP, outside the main direction of prevailing winds and water flow of the region to determine local baseline values.

#### 2.2.1 Processing of soil samples

Soil samples were dried on polyethylene sheets at ambient temperature (25–30 °C) to a constant weight, disaggregated using a porcelain mortar and pestle and screened through a 150 µm nylon mesh to remove organic residues. Thereafter the soil fraction was transferred into airtight zip-lock bags and stored until analysis.^24^

#### 2.2.2 Plant sample processing

Rice plants were separated into root, shoot, and grain components using stainless steel scissors. The root tissues were rinsed repeatedly with Milli-Q water to remove adhered soil particles. All plant tissues were oven-dried at 70 °C for 72 h until constant weight.^25^ Dried tissues were ground in a porcelain mortar and pestle, sieved through a 55 µm nylon mesh and stored in airtight zip lock bags until further analysis.

### 2.3 Chemical analysis of soil samples

Soil pH was determined in a 1 : 2.5 soil : deionized water suspension (10 g soil + 25 mL water) using a calibrated digital pH meter (Cole Parmer P200-02).^26^ A 1 : 5 soil–water extract was used to measure the electrical conductivity (EC) using a conductivity meter (Eutech CON700).^27^ Both measurements were performed in triplicates. Carbon (C%), nitrogen (N%), hydrogen (H%) were quantified using a CHNS elemental analyzer (UNICUBE^®^) with approximately 2–5 mg of finely ground soil combusted for elemental analysis.^28^

### 2.4 Potentially toxic element analysis

#### 2.4.1 Soil digestion

PTE concentration in soil was determined following USEPA Method 3050B.^29^ Briefly, 1.00 g of air-dried, sieved soil was transferred to a digestion vessel and 10 mL of concentrated HNO_3_ (1 : 1 v/v) was added and the mixture was refluxed at 95 °C ± 10 °C for 15 min without boiling. After cooling, an additional 5 mL concentrated HNO_3_ was added, and refluxing continued until brown fumes ceased. The solution was evaporated to approximately 5 mL without boiling. After cooling, 2 mL Milli-Q water and 3 mL 30% H_2_O_2_ were added, and the mixture was heated until effervescence subsided. Additional 1 mL H_2_O_2_ aliquots were added until sample appearance stabilized. The digestate was heated with 10 mL concentrated HCl (reflux, 15 min), diluted to 50 mL with Milli-Q water, and filtered through Whatman No. 42 ashless filter paper. Filtrates were stored in acid-washed polyethylene bottles at 4 °C until analysis.

#### 2.4.2 Plant tissue digestion

Finely ground plant tissues (0.50 ± 0.005 g) were weighed into 100 mL borosilicate beakers. Concentrated HNO_3_ (10 mL) was added and allowed to pre-digest at room temperature for 1 h. The mixture was heated at 80 °C for 30 min, followed by addition of 5 mL HClO_4_. The temperature was increased to 150 °C and maintained until dense white fumes evolved and the solution became clear. Where complete digestion was not achieved, an additional 2 mL HNO_3_ was added and heating continued. The final volume was reduced to approximately 0.5–1 mL, cooled, diluted to 50 mL with Milli-Q water, and filtered through Whatman No. 42 paper. Digests were stored at 4 °C until analysis.^30^

### 2.5 Quality assurance (QA) and quality control (QC)

PTE concentrations in plant tissues and soil samples were analysed using inductively coupled plasma optical emission spectrometry (ICP-OES) (ICAP™ PRO X DUO). QA and QC were rigorously implemented through the analysis of triplicate samples alongside procedural blanks. The reliability of the digestion and analytical methods was validated using a certified reference material (CRM), MESS-4, a marine sediment standard for total and extractable metals sourced from the National Research Council of Canada. The recovery rates for both the CRM and spiked samples were between 98% and 108%. Instrument calibration was carried out using Certipur^®^ ICP Multi-element Standard Solution IV, Certified Reference Material. Calibration curves with *R*^2^ ≥ 0.9969 were accepted. Calibration verification standards were analysed every 10 samples. Procedural blanks were included and blank-corrected values were used for final concentration calculations. Analytical precision was ensured by maintaining relative standard deviations within ±5% for all measured elements. The method detection limits for Cu, Pb, As, Ni, Zn, Co, Cd, and Cr were 0.002, 0.021, 0.003, 0.002, 0.01, 0.0027, 0.015, and 0.002 mg kg^−1^, respectively. For rice plant samples, method accuracy and matrix effects were evaluated through spike recovery analysis, yielding recovery range for PTEs within the 82–121.4% regulatory limit.

### 2.6 Soil pollution indices

Soil contamination by PTEs was evaluated using the Pollution Load Index (PLI), Contamination Factor (CF), Ecological Risk factor (ER), and Potential Ecological Risk Index (PERI). These indices quantify metal enrichment relative to natural background levels and assess anthropogenic influence. The CF compares measured metal concentration with Indian background values to reflect regional geochemical baseline and was calculated as:1
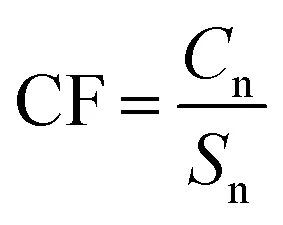
where *C*_n_ is the concentration of metal n in soil and *S*_n_ is the background value (mg kg^−1^). *S*_n_ values used were: As 21.4, Zn 56.9, Pb 66.5, Cd 0.2, Ni 27.7, Cr 114, Cu 56.5 mg kg^−1^, sourced from Indian natural soil background values (INSB).^31^ The overall level of PTE pollution was evaluated through PLI proposed by Tomlinson *et al.*^32^ as:2
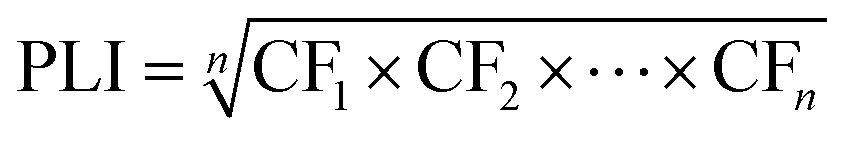
where CF_1_, CF_2_ and CF_*n*_ represent the contamination factors of the individual PTEs and *n* is the number of PTEs considered.

The ecological risk posed by individual metals in the study area was assessed using the ecological risk factor (EF):^33^

3 EF = *T*_r_^*i*^ × CF

where *T*_r_^*i*^ is the toxic response factor (Cd = 30, As = 10, Pb = Co = Ni = Cu = 5, Cr = 2, Zn = 1). The overall Potential Ecological Risk Index (PERI) was calculated as the sum of EF for all metals:4
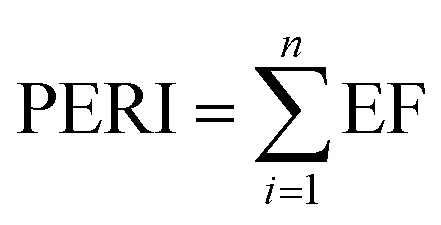


Classification criteria for CF, PLI, EF, and PERI are provided in Table S1.

#### 2.6.1 Bioconcentration and transfer of PTEs from soil to rice

To assess PTE accumulation and translocation efficiency in the rice plants, the bioconcentration factor (BCF) was calculated separately for the root (BCF_R_), shoot (BCF_S_), and grain (BCF_G_) according to Kumar *et al.*:^34^5
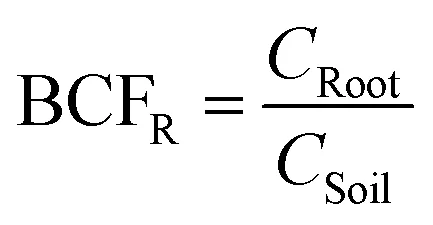
6
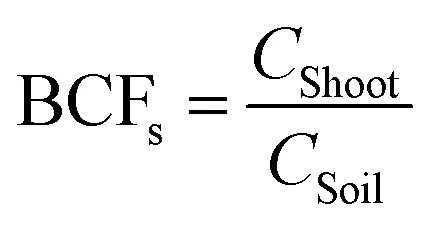
7
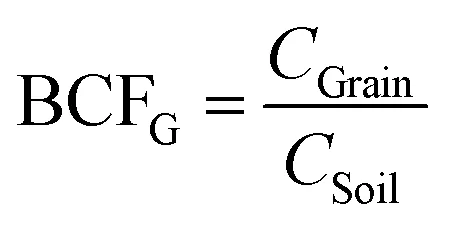
where *C*_Root_, *C*_Shoot_ and *C*_Grain_ represent the PTE concentrations (mg kg^−1^) in roots, shoots, and grains, respectively, and *C*_Soil_ represents the corresponding PTE concentration in soil at each sampling site. A BCF > 1 in any plant tissues indicates effective accumulation of PTEs, whereas BCF < 1 reflects limited uptake from soil.

PTE translocation within the plant was evaluated using translocation factor (TF), calculated as described by Bashir *et al.*:^35^8
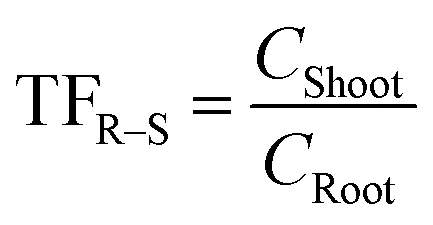
9
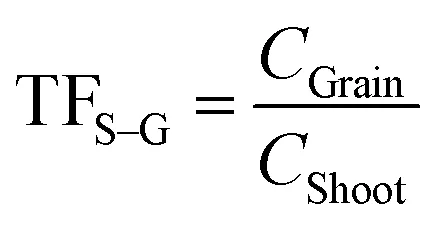
10
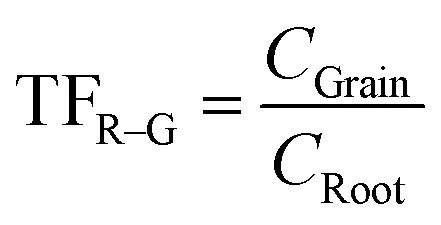
where *C* indicates concentration of PTE, TF_R–S_ indicates the root-to-shoot translocation factor, representing the efficiency of PTE translocation from root to shoot. TF_S–G_ indicates the shoot-to-grain translocation factor, representing the efficiency of PTE translocation from shoots to grains. TF_R–G_ indicates the root-to-grain translocation factor, representing metal transfer from roots to edible grain. A TF values >1 indicates efficient internal metal transport, whereas TF < 1 denotes restricted metal mobility within the plant.^36^

### 2.7 Rice grain contamination indices

#### 2.7.1 Single-element pollution index

The extent of PTE contamination in rice grain was evaluated using the metal pollution index (MPI) following the method described by Zhao *et al.*^37^ The MPI compares the measured concentration of each PTE in rice grain with its corresponding maximum allowable concentration. The index was calculated using eqn (11):11
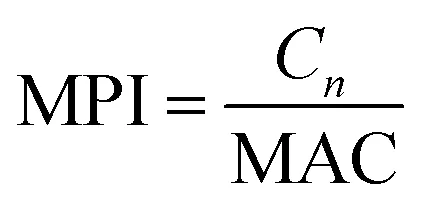
where *C*_*n*_ represents the concentration of a given PTE in crop samples (mg kg^−1^), and MAC denotes the maximum allowable concentration (MAC) (mg kg^−1^) for that element. MAC values for PTEs in food grains used in this study were adopted from the Food Safety and Standards (Food Products Standards and Food Additives) Regulations, 2011, with limits of As (1.1 mg kg^−1^), Zn (50 mg kg^−1^), Pb (0.2 mg kg^−1^), Cd (0.4 mg kg^−1^), Ni (1 mg kg^−1^), Cr (1 mg kg^−1^), and Cu (30 mg kg^−1^).^38^

#### 2.7.2 Composite pollution index

The overall level of PTE contamination in rice grain samples was assessed using the Grain Pollution Factor (GPF). This index was calculated as the geometric mean of individual PTE concentrations in the grains, following Kumar *et al.*:^34^12
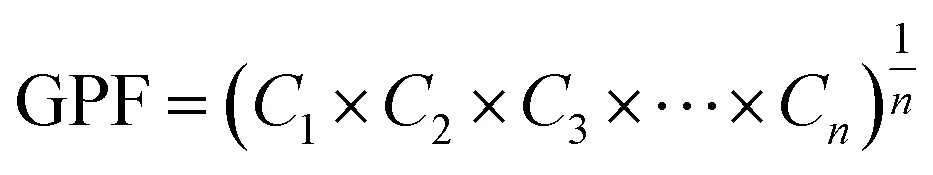
where *C*_*n*_ denotes the concentration of the *n*th PTE in the grain sample (mg kg^−1^). GPF values were classified as: low level pollution (GPF < 1), moderate level pollution (1 ≤ GPF < 3), and high/severe level of pollution (GPF ≥ 3).

### 2.8 Human health risk assessment

Human health risk assessment (HRA) was conducted following the United States Environmental Protection Agency framework,^39^ to evaluate potential health risks associated with the dietary exposure of PTEs through consumption of contaminated rice grains. Health risks were categorized into non-carcinogenic risk (NCR), which reflects adverse health effects from long-term dietary exposure, and carcinogenic risk (CR), which indicates the lifetime probability of developing cancer following exposure to carcinogenic PTEs.^40^

Given that rice is a major dietary staple food in the study area, the assessment focused exclusively on oral ingestion as the primary exposure pathway. Health risks were evaluated for children and adults to account for differences in rice consumption rates, body weight, and physiological sensitivity.^41^ Children were represented using a body weight of 32.5 kg and a 6-year exposure duration, corresponding to early childhood (1–6 years), whereas adults were represented using a body weight of 60 kg and a 70-year exposure duration, corresponding to standard lifetime-average adult exposure assumptions (≥18 years). The average daily dose (ADD) for rice ingestion was calculated using eqn (13):13
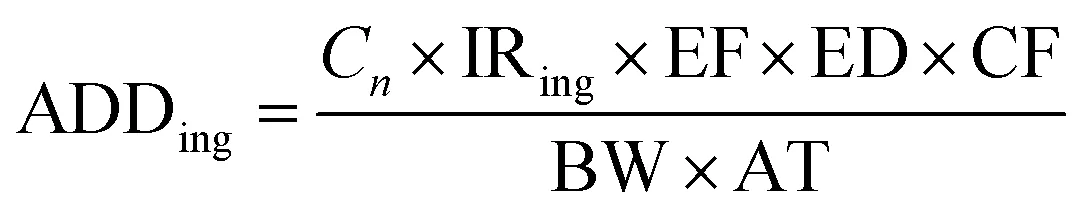
where IR_ing_ is the ingestion rate of rice (kg per day), EF is the exposure frequency (days per year), ED is the exposure duration, BW is body weight, and AT is the averaging time (days). In this study, metal concentration (*C*) and IR_ing_ (kg per day) combine directly to mg per day; consequently, the unit conversion factor CF is equal to 1. Parameter distributions are given in Tables S3–S5. For adults, exposure duration was set equal to lifetime averaging time, such that AT for non-carcinogenic and carcinogenic risk coincide (365 × 70 days); for children, ED = 6 years reflects the assumed childhood exposure period, with AT for non-carcinogenic risk calculated as 365 × 6 days.

Non-carcinogenic risk was quantified using the hazard quotient (HQ) by comparing ADD with the reference dose (RfD), and hazard index (HI) was calculated as the sum of HQs for all metals:14
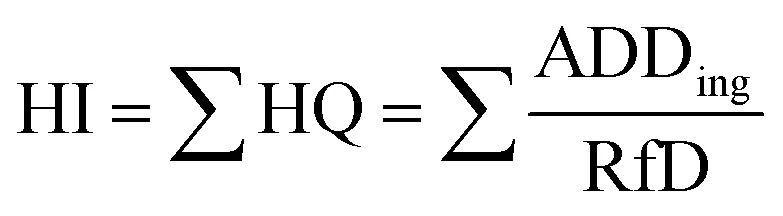
where RfD is the reference dose for each metal (Table S5). Carcinogenic risk was estimated using the CR for individual metals, and the Total Carcinogenic Risk (TCR) was calculated by adding CR values across all carcinogenic metals as:

15 TCR = ∑CR = ∑(ADD × SF)

where SF is the oral slope factor (kg day mg^−1^) for each metal, obtained from USEPA and regional literature sources (Table S5). To account for variability and uncertainty in exposure parameters, a probabilistic health risk assessment was performed using Monte Carlo simulation with 10 000 iterations. Results were summarized using mean values and cumulative distribution functions, and uncertainty ranges were expressed using percentile statistics of the simulated outputs. This approach provides a more realistic evaluation of dietary health risks by incorporating probability distributions for key input variables.^42^ The deterministic parameter values and probability density function settings used in the simulations are presented in Tables S2–S5.

### 2.9 Statistical analysis

Basic statistical analyses were performed by Microsoft Excel 2021 (Microsoft, USA) and PAST software (version 4.03). Data normality was assessed using the Shapiro–Wilk test, revealing non-normal distributions for most metals in soil, root, shoot, and grain samples (*p* < 0.05). Consequently, non-parametric tests were employed. Differences in metal concentrations across matrices were evaluated using the Kruskal–Wallis test at significance level of *α* = 0.05. Principal component analysis (PCA) and graphical visualizations were generated using Origin-Pro 2025b. Human health risk assessment was performed in MATLAB 2025b using Monte Carlo simulations. ArcGIS version 10.8 was used to generate georeferenced spatial maps using the WGS 1984 coordinate system and to integrate sampling location data into the spatial database. The spatial distribution of variables was estimated using the Inverse Distance Weighted (IDW) interpolation method with a power parameter of 2. This approach predicts unknown values based on the distance-weighted average of surrounding measured data points.^43^

## 3 Results

### 3.1 Chemical properties of soil

The agricultural soil in the study area was predominantly slightly alkaline (pH: 7.91 ± 0.3) with low to moderate salinity (EC: 383.27 ± 10.3 µS cm^−1^) and moderate organic matter content, as indicated by total carbon (1.45%) and nitrogen (0.12%), while organic carbon was 2.58 ± 0.52% (Table 1). The coefficient of variation (CV) indicated relatively low variability for pH, EC and moderate variability for C, N and H. Kurtosis values ranged between −1.0 and 0.9, indicating relatively flatter distributions compared to normal distribution.

**Table 1:** Descriptive statistics of soil physicochemical properties in the study area^a^

	pH	EC (µS cm^−1^)	N (%)	C (%)	H (%)	CEC (cmol(+) kg^−1^)	OC (%)
Average	7.9	383.3	0.1	1.5	1.3	29.8	2.58
Min	6.9	224	0.12	1.1	0.7	18.2	1.89
Max	8.4	612	0.2	2	1.8	42.6	3.44
SD	0.3	10.3	0.008	0.3	0.3	5.8	0.52
CV%	3.8	2.7	6.7	20.0	23.0	19.4	20.1
Kurt	0.9	−0.9	−1	−0.4	0.4	−0.3	−0.4
BV	7.8	307.3	0.02	0.3	0.6	26.4	0.52

a BV: background value.

The Shapiro–Wilk test indicated significant deviations from normality for most parameters (*p* < 0.05), justifying the application of non-parametric statistical approaches. Accordingly, the buffer zone-specific spatial variations were analysed using the Kruskal–Wallis test. The results showed no statistically significant variation in pH, N, C or hydrogen (*p* > 0.05), whereas EC exhibited significant spatial heterogeneity (*p* < 0.05), suggesting localized differences in soil salinity conditions within the study area.

### 3.2 Concentration and distribution pattern of PTEs in paddy soil

**Fig. 2:**
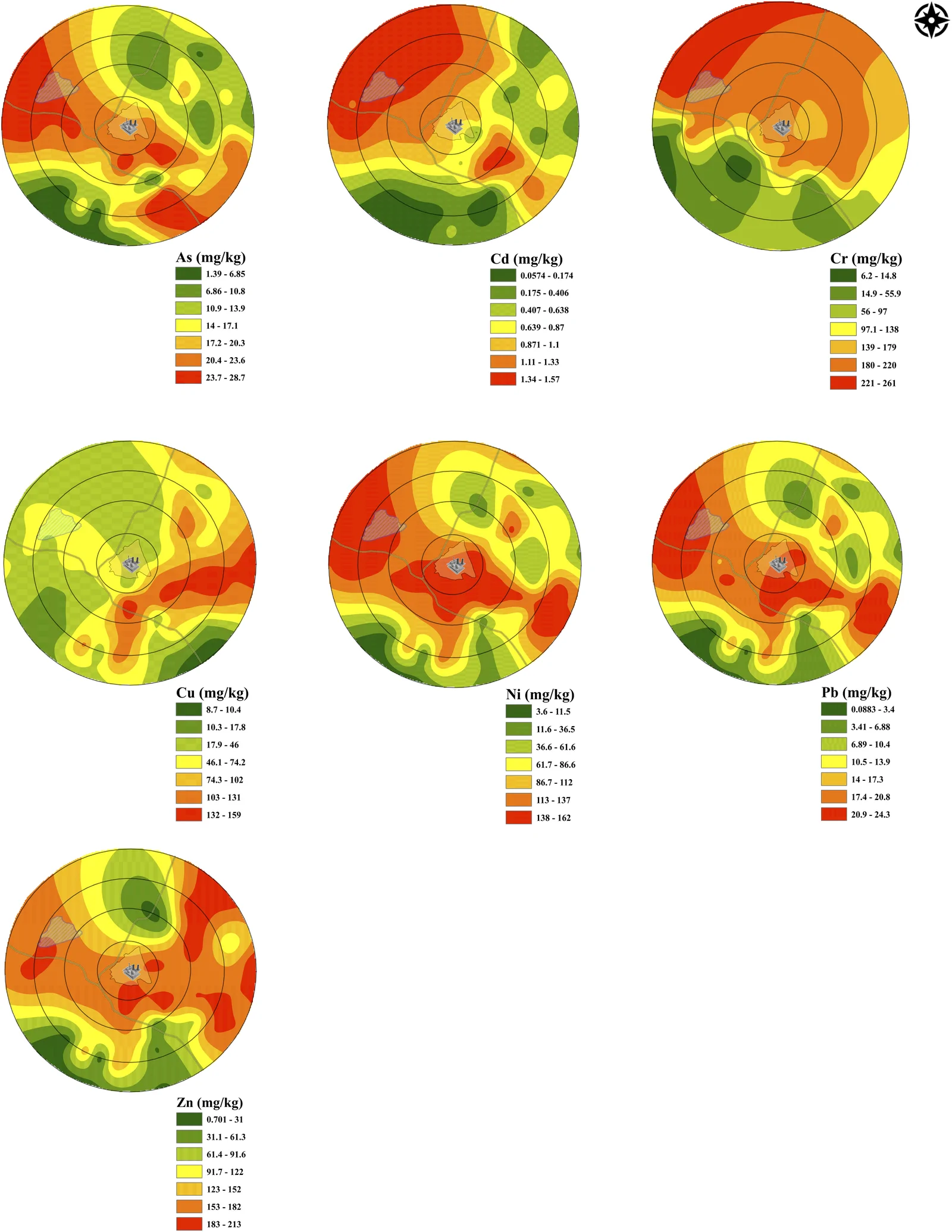
Spatial distribution of potentially toxic elements in paddy soils across the study area.

The concentration of PTEs in the agricultural soils in the proximity of TTP varied, having the mean descending sequence of Cr (134.7) > Zn (128.8) > Ni (105.7) > Cu (98.0) > As (16.8) > Pb (15.8) > Cd (1.0) (mg kg^−1^) (Table 2). Spatial mapping of PTEs distribution showed clear heterogeneous patterns within the study area (Fig. 2). Cr and Ni exhibited relatively extensive enrichment surrounding the TTP and the associated fly ash pond. Cr concentrations formed a relatively diffuse, wide plume stretching northward toward the highway on the northern side of the pond, whereas Ni exhibited elevated levels forming an east–west elongated belt of contamination in the agricultural areas on both sides of TTP. Concentrations of As formed a distinct, narrow plume extending east-to-west, starting close to the ash pond and the main facility buildings with concentrations decreasing with increasing distance from the sources. Cd showed a more localized distribution, including a distinct hotspot west of the plant near the periphery of the ash-disposal area. Cu was mainly enriched in the east, especially in the more intensively cultivated rice paddies. Zn showed enrichment in the northeast area and the central area close to TTP, while Pb showed the most scattered distribution with some east–west enrichment in the area and no major hotspot.

**Table 2:** Concentration of PTEs in soil samples with background and guideline limits^a^

	As	Zn	Pb	Cd	Ni	Cr	Cu
Average	16.8	128.8	15.8	1.0	105.7	134.7	98.0
Min	3.8	22.4	2.5	0.1	18.2	23.8	21.5
Max	26.3	198.5	24.3	1.5	160.3	205.0	140.2
SD	6.6	60.0	7.1	0.5	47.9	61.7	36.8
CV%	39.3	46.6	44.9	50.0	45.3	45.8	37.6
Kurt	−1.1	−1.3	−1.2	−1.1	−1.3	−1.3	−0.8
BV	21.4	56.9	66.5	0.2	67.8	109.4	73.2
ILS	5	250	5	1	20	5	25
INSB	—	22.1	13.1	0.2	27.7	114	56.5
WHO	—	50	85	0.8	35	100	36
CCME	12	200	70	1.4	50	64	91

a ILS: Indian limits of soil:^34^ INSB: Indian natural soil background:^31^ BV: background value; WHO: World Health Organization^45^ CCME: Canadian Council of Ministers of the Environment.^44^

Comparison with background values and regulatory standards revealed varying degrees of exceedance (Table 2). Mean concentrations of Cr and Ni showed the most critical enrichment, with concentrations exceeding the Canadian Council of Ministers of the Environment (CCME) guidelines indicating elevated levels in the agricultural soils. Mean Cu concentrations also exceeded both the CCME guideline^44^ and the Indian limit of soil (ILS) standard.^34^ As remained below the local background value but surpassed the CCME guideline. Cd mean value was within CCME and ILS limits, yet its concentration was five times higher than the reported background values. Pb mean values remained below the CCME guideline but exceeded the ILS while Zn, although approximately 2.3 times greater than the background concentration, remained within the CCME permissible limit.

### 3.3 Pollution assessment for soil

#### 3.3.1 Contamination factor and pollution load index

**Fig. 3:**
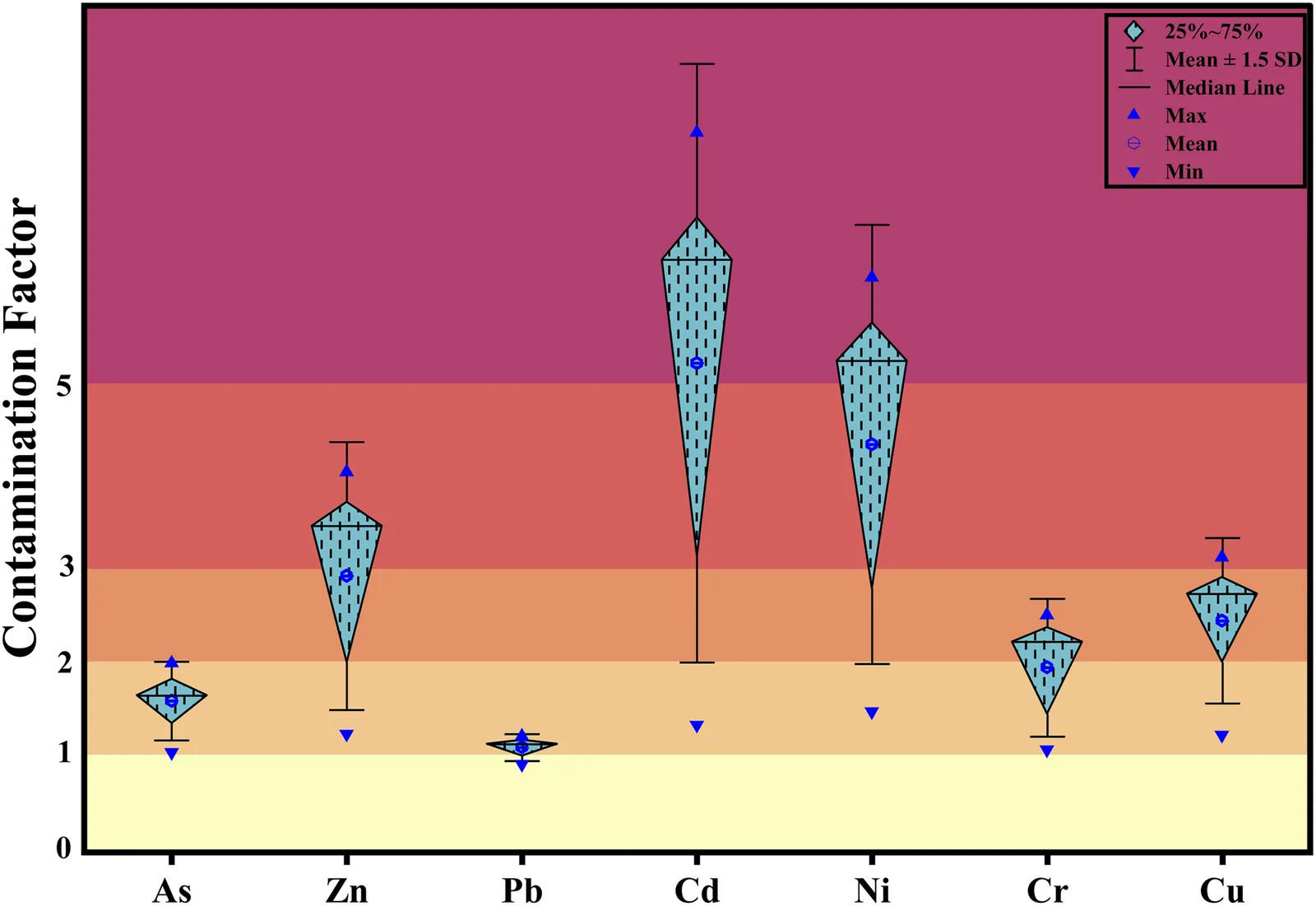
Contamination factor of potentially toxic elements in paddy soil samples.

Enrichment patterns specific to individual metals were observed across the study area (Fig. 3 and Table S9). Cd exhibited the highest CF values, ranging from 0.50 to 7.50. Among the 47 sampling sites, 35 sites (74.5%) had CF values ≥3, including 7 sites (14.9%) in the high contamination category (3 ≤ CF < 5) and 28 sites (59.6%) in the extremely high contamination category (CF ≥ 5), while 9 sites (19.1%) showed moderate contamination and 3 sites (6.4%) showed low contamination. Cd therefore represented one of the major contaminating metals in the study area. In addition, substantial contamination was associated with Zn and Ni. The CF for Zn ranged from 0.39 to 3.49, with 29 sites (61.7%) showing moderate contamination (2 ≤ CF < 3) and 18 sites (38.3%) showing high contamination (3 ≤ CF < 5). The CF values for Ni ranged from 0.66 to 5.79, with 36 sites (76.6%) showing CF values ≥2; among these, 31 sites (66.0%) were classified as highly contaminated (3 ≤ CF < 5) and 21 sites (44.7%) as extremely highly contaminated (CF ≥ 5). These results indicate that Ni was also a major contaminant and exhibited substantial enrichment across the study area.

Cu and Pb generally showed lower CF values than Cd, Zn, and Ni. The CF values for Cu varied from 0.38 to 2.48, with 24 sites (51.1%) showing moderate contamination (2 ≤ CF < 3), 14 sites (29.8%) showing low contamination (1 ≤ CF < 2), and 9 sites (19.1%) showing non-pollution (CF < 1). Pb had the lowest CF values among all the metals studied, ranging from 0.04 to 0.37, with all 47 sites (100%) showing CF values below 1, indicating non-pollution with respect to Pb. The metals As and Cr also showed comparatively lower CF values. The CF values for As ranged from 0.18 to 1.23, with 33 sites (70.2%) showing non-pollution and 14 sites (29.8%) showing low contamination. Cr exhibited CF values ranging from 0.21 to 1.80, with 17 sites (36.2%) showing non-pollution and 30 sites (63.8%) showing low contamination.

**Fig. 4:**
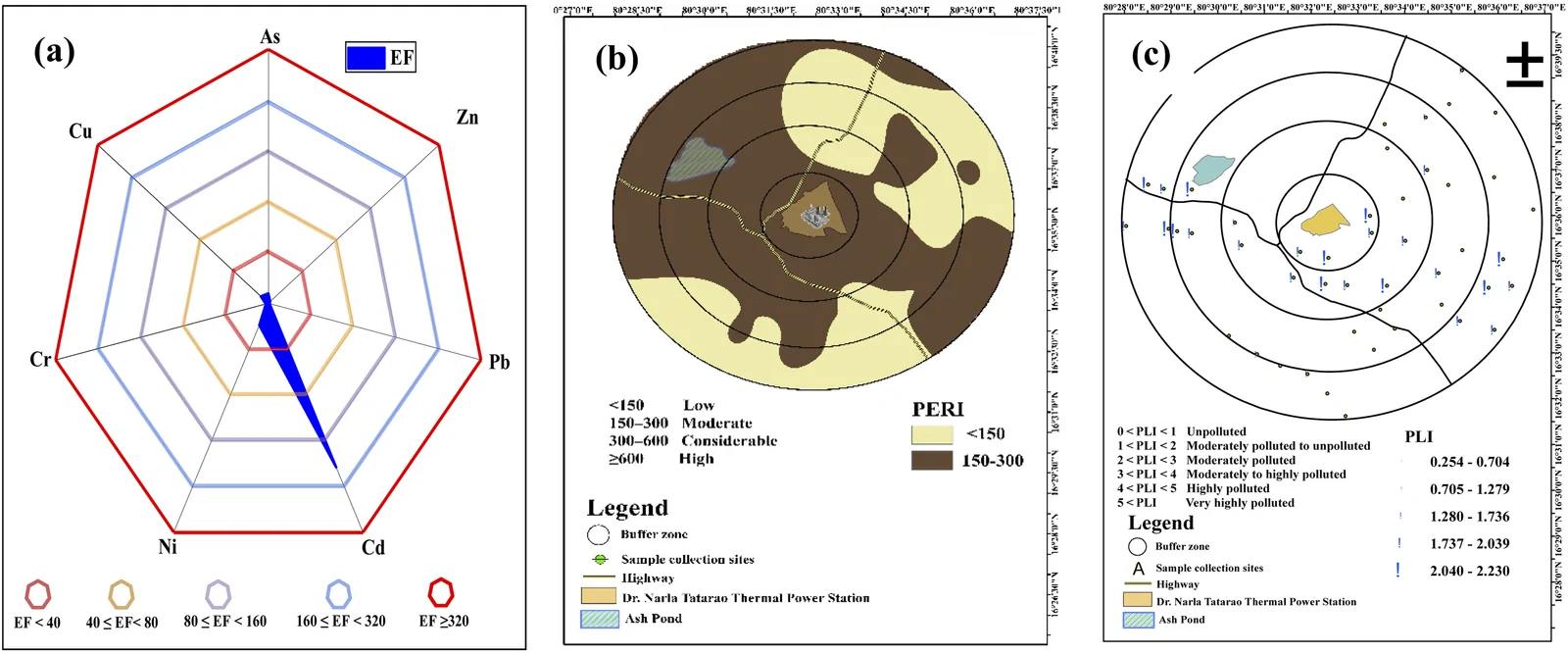
(a) Ecological risk factor (EF), (b) potential ecological risk index (PERI), and (c) pollution load index (PLI) of PTEs in paddy soil.

PLI, representing the cumulative effect of all analysed metals, ranged from 0.25 to 2.23, with an average PLI of 1.48 ± 0.65 (Table S9). Of the 47 sampling sites, 14 sites (29.8%) had PLI values <1, 21 sites (44.7%) had PLI values between 1 and <2, and 12 sites (25.5%) had PLI values ≥2, indicating a transition from unpolluted to moderately/heavily polluted conditions across the study area. Overall, 33 sites (70.2%) had PLI values ≥1, indicating the presence of composite pollution at a substantial proportion of the sampling locations. Spatially, higher PLI values were observed in the belt between the thermal power plant and ash pond and along the roads on either side of the thermal power plant, particularly toward the eastern direction (Fig. 4c).

#### 3.3.2 Ecological risk factor and potential ecological risk index for soil PTEs

The ecological risk posed by the PTEs in the soil was assessed using the ecological risk factor (EF) of each PTE and the integrated potential ecological risk index (PERI). The mean EF values revealed a clear hierarchy of ecological risk among the analysed elements (Fig. 4a). Cd exhibited the highest mean ER (143.30), followed by Ni (19.08), Cu (8.67), As (7.87), Cr (2.36), Zn (2.26), and Pb (1.19). Thus, Cd represented the dominant contributor to individual ecological risk, followed by Ni. The ER values for As, Zn, Pb, Ni, Cr, and Cu remained below 40 across the study sites, corresponding to a low ecological risk category. In contrast, Cd showed substantially higher ER values, ranging from 15.0 to 225.0, indicating that its ecological risk varied from low to considerable across the sampling locations (Table S10).

The integrated PERI values, representing the cumulative ecological risk from all analysed metals, ranged from 22.96 to 286.65, with a mean value of 184.73 ± 88.24. Based on the standard classification criteria (Table S1), 15 sites (31.9%) were classified as having low potential ecological risk, whereas 32 sites (68.1%) exhibited moderate potential ecological risk. No sampling site fell within the considerable or high potential ecological risk categories. The relatively higher PERI values were concentrated in the northwestern and central-eastern sectors, coinciding with areas exhibiting elevated soil metal concentrations (Fig. 4b).

### 3.4 PTE partitioning in rice plant tissues

**Fig. 5:**
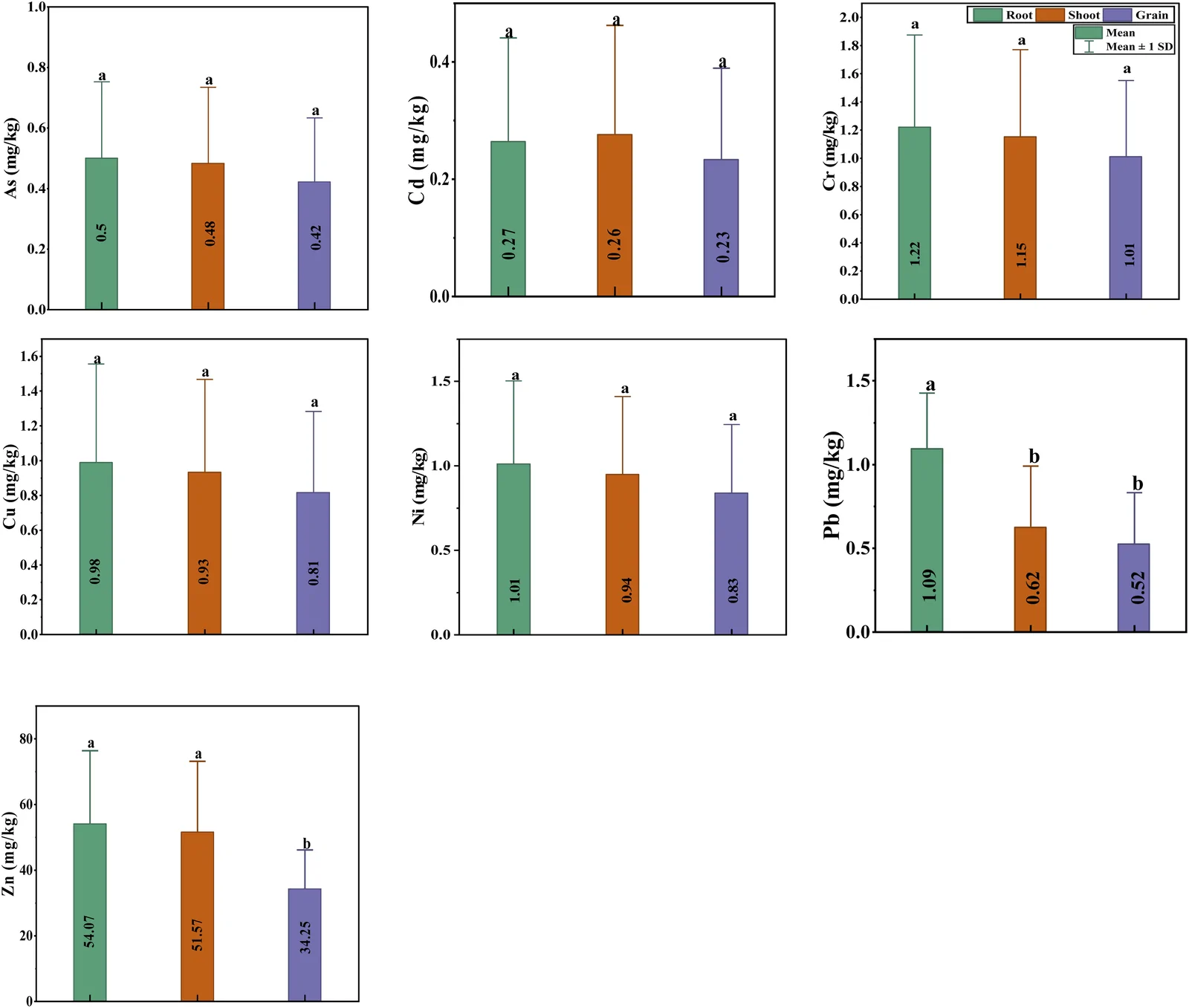
Partitioning of PTEs in different parts of the rice plant, including root, shoot, and grain.

The accumulation of PTEs in rice tissues showed distinct differences between root and shoot compartments (Fig. 5). Among all analysed PTEs, Zn exhibited the highest accumulation in both roots and shoots, with mean concentrations exceeding 51 mg kg^−1^. The concentrations of As, Pb, and Cr were higher in roots than in shoots, indicating greater retention in belowground tissues. Pb particularly showed elevated root accumulation, with a mean concentration of 1.09 mg kg^−1^ compared to 0.62 mg kg^−1^ in shoots. In contrast, Cd and Ni showed comparable or slightly higher mean concentrations in shoots than in roots. Zn and Cu exhibited relatively similar mean concentrations in both tissues.

Analysis of the rice grains revealed a distinct pattern of PTE accumulation, with mean concentrations descending in the order: Zn (34.26) > Cr (1.01) > Ni (0.84) > Cu (0.82) > Pb (0.52) > As (0.42) > Cd (0.23) mg kg^−1^. For all analysed PTEs, the mean grain concentration was lower than the corresponding mean in both root and shoot tissues, indicating comparatively lower accumulation in the edible grain compartment.

### 3.5 Bioaccumulation patterns in rice plant tissues

**Fig. 6:**
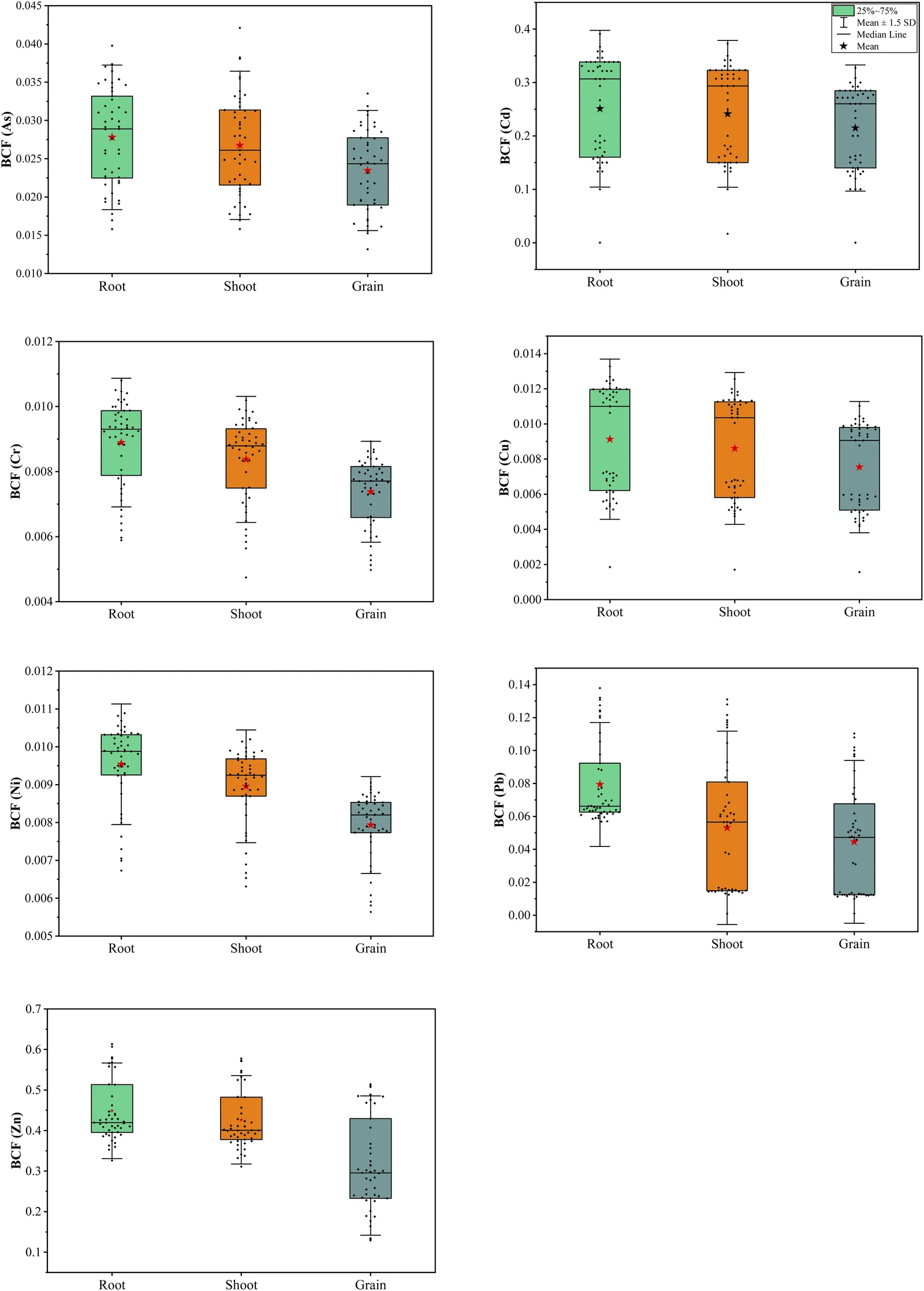
Bioconcentration factor (BCF) showing variation in metal accumulation among different parts of the rice plant (root, shoot, and grain).

The BCF revealed clear PTE-specific patterns of uptake from soil into rice tissues (Fig. 6 & S1). For roots, mean BCF values were below 1 for all PTEs, indicating generally limited accumulation relative to soil concentrations. Among the analysed elements, Cd exhibited comparatively higher root uptake (mean BCF_R_ ≈ 0.25), while Zn also showed relatively elevated root accumulation. In contrast, Cr and Cu recorded the lowest root BCF values (BCF_R_ ≈ 0.01), suggesting strong exclusion at the root–soil interface. Overall, the root compartment demonstrated greater bioaccumulation than the aerial tissues. Mean BCF_S_ values were generally lower than those of roots, suggesting restricted upward movement after initial uptake. Pb exhibited particularly low shoot BCF_S_ values (mean ≈ 0.03), whereas Cd and Zn showed comparatively higher BCF_S_ values relative to other PTEs, although all remained below 1. Accumulation in rice grains was the most restricted among all plant compartments. BCF_G_ values were consistently the lowest, with mean values ranging from approximately 0.007 for Cr to 0.21 for Cd. Notably, As and Cd showed comparatively higher BCF_G_ values than other metals. In contrast, Pb, Cr, and Cu exhibited low grain accumulation.

### 3.6 Translocation pattern in rice plant tissues

**Fig. 7:**
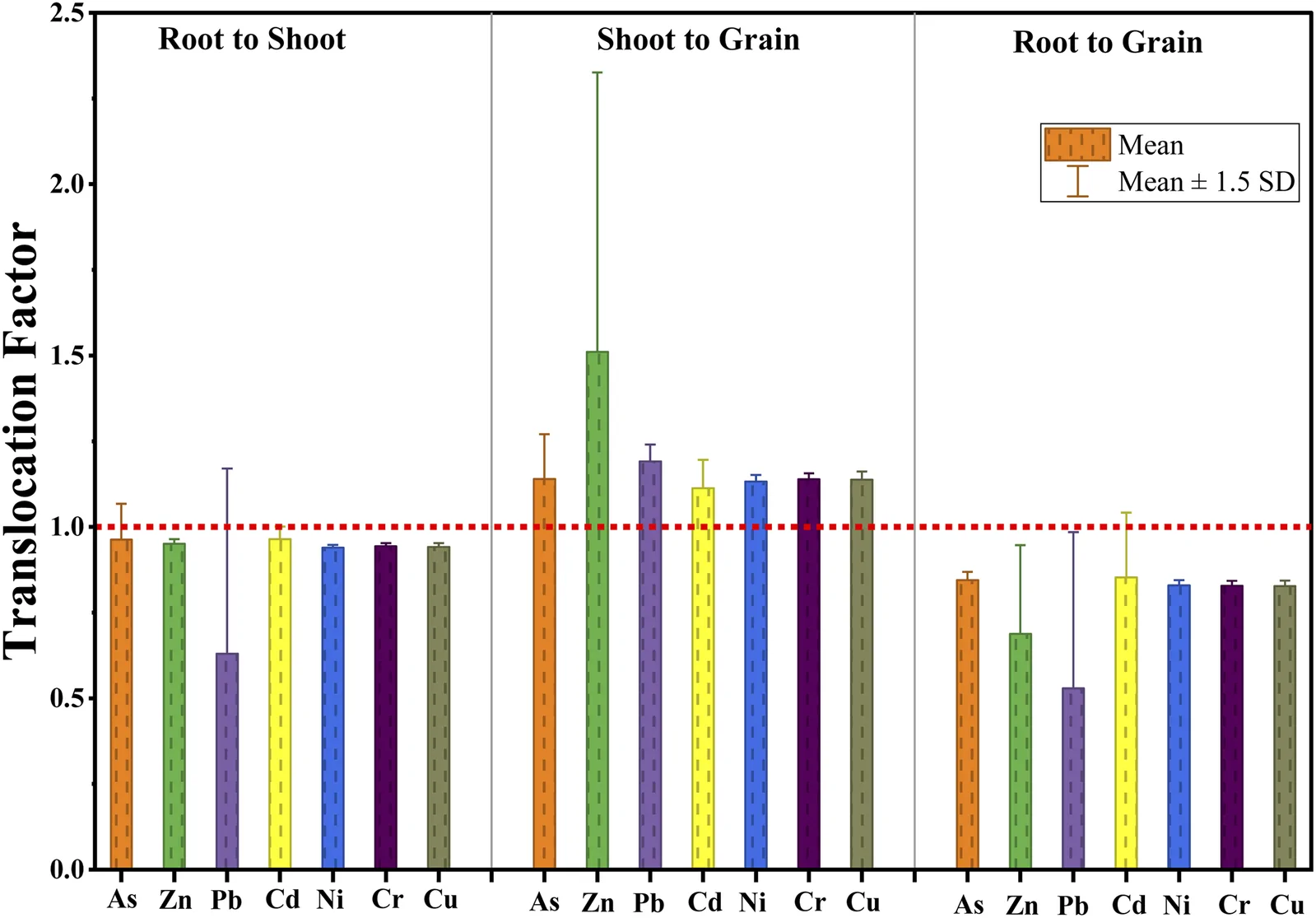
Translocation factor of potentially toxic elements showing the transfer of elements within rice plants, including root-to-shoot, shoot-to-grain, and root-to-grain.

The TF was used to evaluate the internal mobility of PTEs between plant tissue compartments (Fig. 7). The root-to-shoot TF values were predominantly below 1 across most sampling sites, indicating greater retention in the root compartment. However, distinct differences were observed among elements. Cd and Zn exhibited comparatively higher root-to-shoot TF values, frequently approaching unity, indicating relatively greater mobility within the plant system. In contrast, Pb demonstrated the most restricted translocation, with root-to-shoot TF values as low as 0.011 at certain sites. Translocation from shoot to grain was considerably more limited. Shoot-to-grain TF values were generally low, often below 0.2. Although Cd showed relatively higher mobility within vegetative tissues, its transfer to grain remained suppressed compared to root and shoot compartments. As and Zn displayed slightly higher shoot-to-grain TF values than other metals, whereas Pb and Cr exhibited negligible movement into grains, consistent with their low grain concentrations. The composite root-to-grain TF values were uniformly low for all metals, typically with mean values below 0.25. Cd showed the highest relative potential for complete soil-to-grain transfer, while Pb and Cr exhibited the lowest.

### 3.7 Pollution assessment for rice grain

#### 3.7.1 Grain pollution factor

The GPF values ranged from 0.11 to 1.64, with a mean of 1.03 (±0.46), indicating variable degrees of metal contamination in rice grains across the study area. Based on the established classification criteria, 72.3% of the sites had GPF values ≥1 and were classified as showing moderate pollution, whereas 27.7% had GPF values <1 and were classified as showing low pollution. None of the sampling locations exceeded the threshold for severe pollution (GPF > 3).

**Fig. 8:**
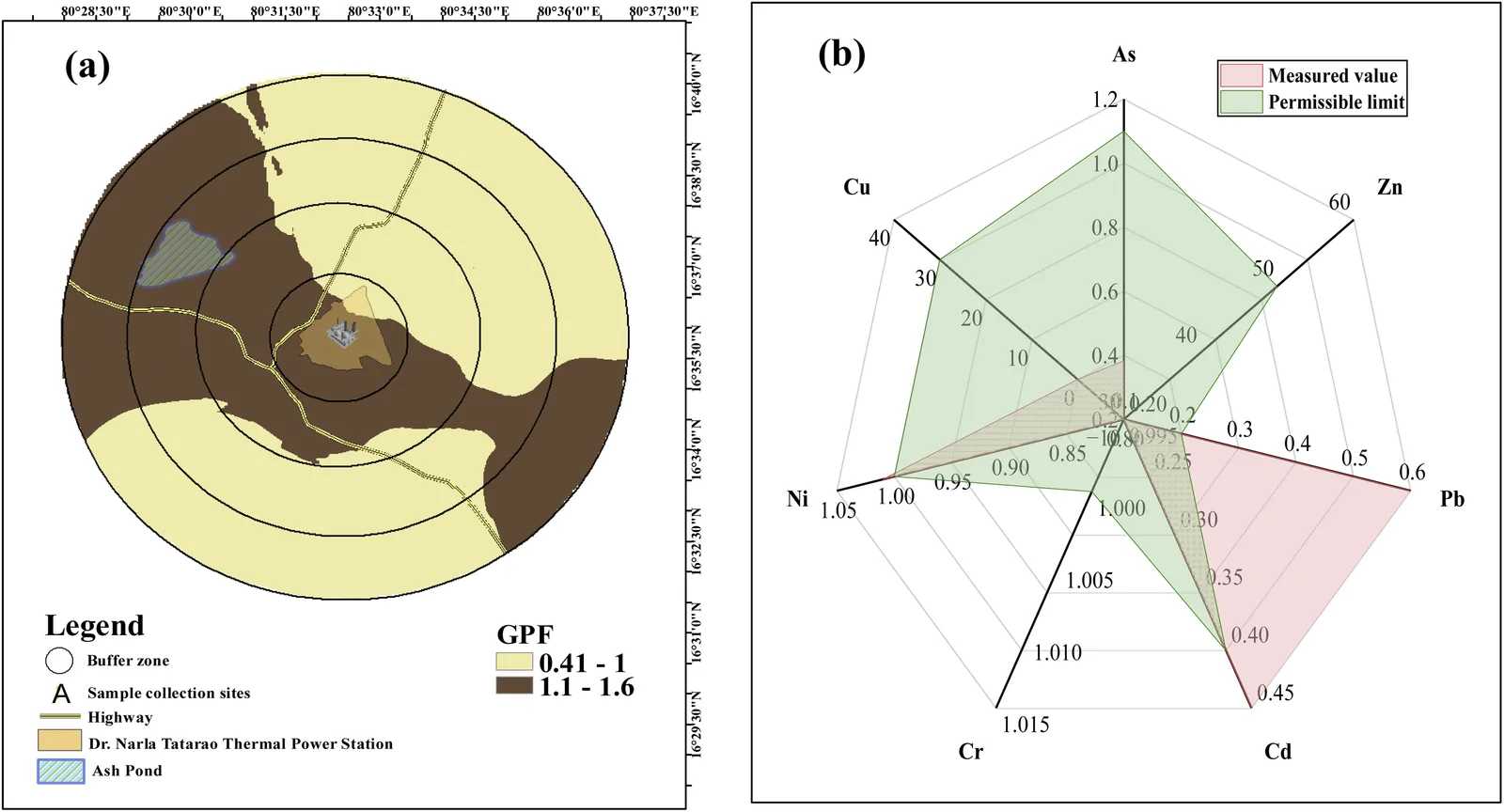
(a) Grain pollution factor (GPF) and (b) metal pollution index of PTEs in rice grains across the sampling sites.

The spatial distribution map of GPF (Fig. 8a) further illustrates a distinct contamination pattern. Higher GPF values (≥1.5) formed a pronounced central belt concentrated around the thermal power plant and ash pond, extending eastward into adjacent agricultural fields. In contrast, comparatively lower GPF values were observed in peripheral areas, indicating lower composite PTE accumulation in rice grains farther from the main plant-associated area.

#### 3.7.2 Metal pollution index

The contamination status of rice grains was evaluated using the MPI, calculated as the ratio between measured PTE concentration and the corresponding FSSAI maximum allowable concentration (Fig. 8b and Table 3). Pb emerged as the most critical contaminant among the analysed PTEs with MPI values ranging from 0.05 to 5.70. A total of 66% of the sites exceeded the permissible limit (MPI ≥ 1), indicating widespread Pb contamination in rice grains. Severe pollution (MPI > 3) was recorded at 7 sites, with the highest values observed at sites 16 (5.65), 40 (5.70), and 42 (5.60), reflecting concentrations more than 5.5 times the allowable limit. Cr and Ni were also prevalent contaminants. The MPI for Cr ranged from 0.11 to 1.53, exceeded the safety threshold at 72% of the sites, making it the most frequently exceeded metal in terms of site count. For Ni, MPI values ranged between 0.16 and 1.23; with 66% sites recorded values above the permissible limit. Zn showed comparatively moderate exceedances, with MPI between 0.23 to 1.09 and 34% sites surpassing the standard. In contrast, Cd, As, and Cu consistently remained within safe limits across all locations. The MPI for Cd ranged from 0 to 0.98, for As from 0.05 to 0.58, and for Cu from 0.003 to 0.042. Overall, the MPI results identified Pb as the PTE with the greatest magnitude of exceedance, while Cr showed the highest frequency of exceedance, followed by Pb and Ni.

**Table 3:** Statistical summary of the metal pollution index (MPI) for PTEs in rice grains

Parameter	As	Cd	Cr	Cu	Ni	Pb	Zn
MPI range (min–max)	0.05–0.58	0.00–0.98	0.11–1.53	0.003–0.042	0.16–1.23	0.05–5.70	0.23–1.09
Mean MPI (±SD)	0.39 ± 0.18	0.59 ± 0.37	1.02 ± 0.54	0.03 ± 0.01	0.86 ± 0.34	2.31 ± 1.60	0.70 ± 0.25
% Sites exceeding (MPI ≥ 1)	0%	0%	72%	0%	66%	66%	34%

### 3.8 Source of metal contributions in soil and rice tissues

**Fig. 9:**
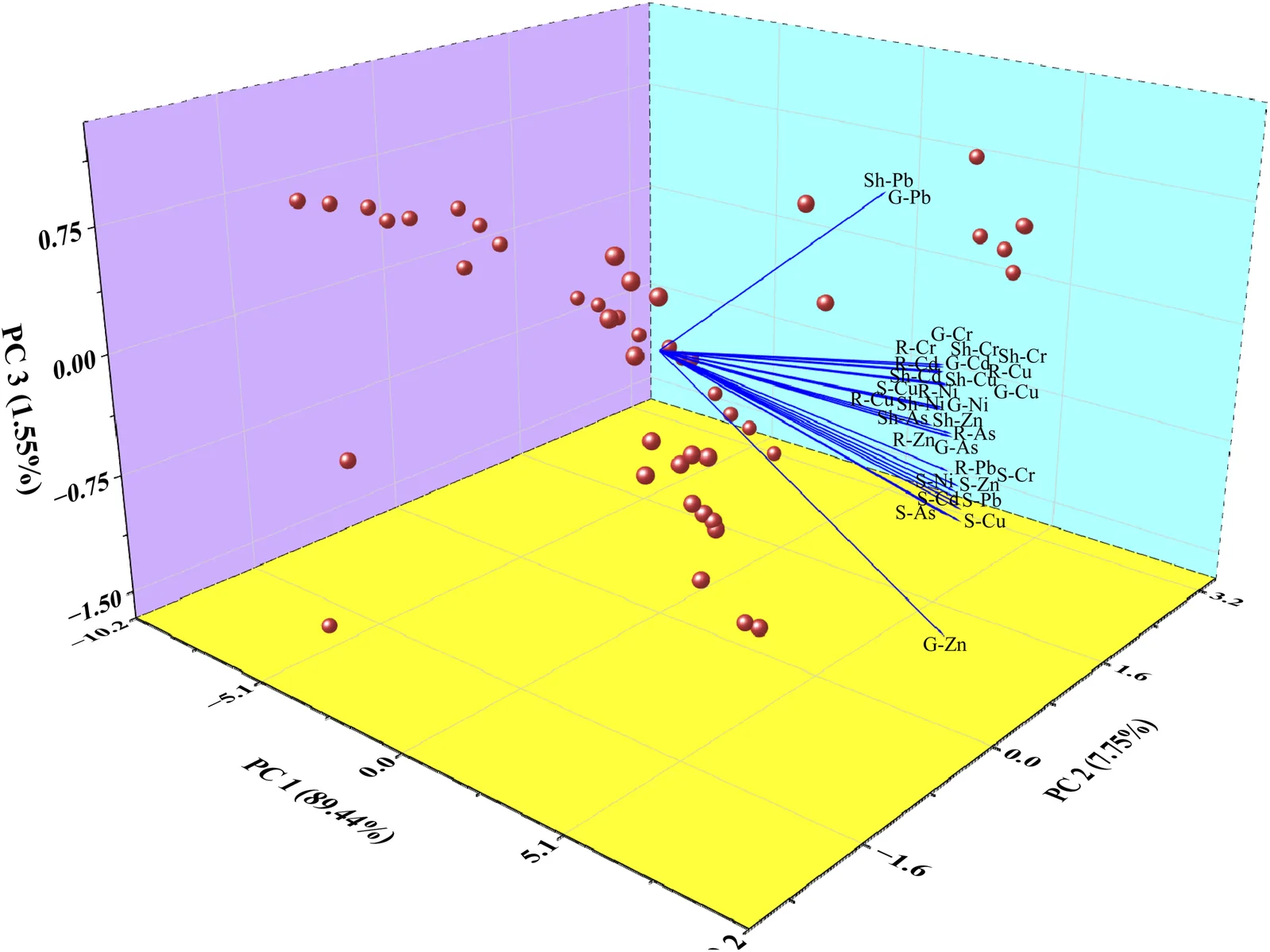
Three-dimensional PCA biplot of PTE concentrations in soil, root, shoot and grain compartments. Arrows represent variable loadings, indicating the relative contribution and direction of each metal, while points denote individual sampling sites. S, R, Sh and G represent soil, root, shoot and grain, respectively; the complete list of abbreviations is provided in Table S13.

PCA was performed on the dataset consisting of PTE concentrations in soil, root, shoot, and grain tissues to identify common patterns of covariance and potential source-related associations (Fig. 9). Prior to analysis, all data were standardized to remove scale effects. The PCA extracted 27 principal components (PCs). PCs were retained based on the Kaiser criterion (eigenvalues > 1), indicating statistically meaningful contributions to total variance (Table S11). Source interpretation was performed by examining PTEs with strong factor loadings (|loading| ≥ 0.5) within the retained components, which were considered significant contributors to the corresponding source profiles. The first principal component (PC1) accounted for 89.4% of the total variance in the dataset. The second component (PC2) explained an additional 7.75% of the variance. Collectively, the first two PCs explained 97.1% of the total variance, with the remaining components each contributing less than 2% individually and were considered insignificant for further interpretation.

The loadings of the original variables on the first two principal components are summarized in Table S12. In PC1, all measured variables showed consistent positive loadings of approximately 0.20, below the adopted threshold. PC2 was primarily associated with Pb in shoot and grain tissues, both showing loadings of 0.671, suggesting a distinct association of Pb between these two compartments. Soil Pb (0.054) and Cd across shoot (−0.042), root (−0.039), and grain (−0.037) showed negligible contributions to PC2.

### 3.9 Influence of soil properties on PTE bioaccumulation in rice

**Fig. 10:**
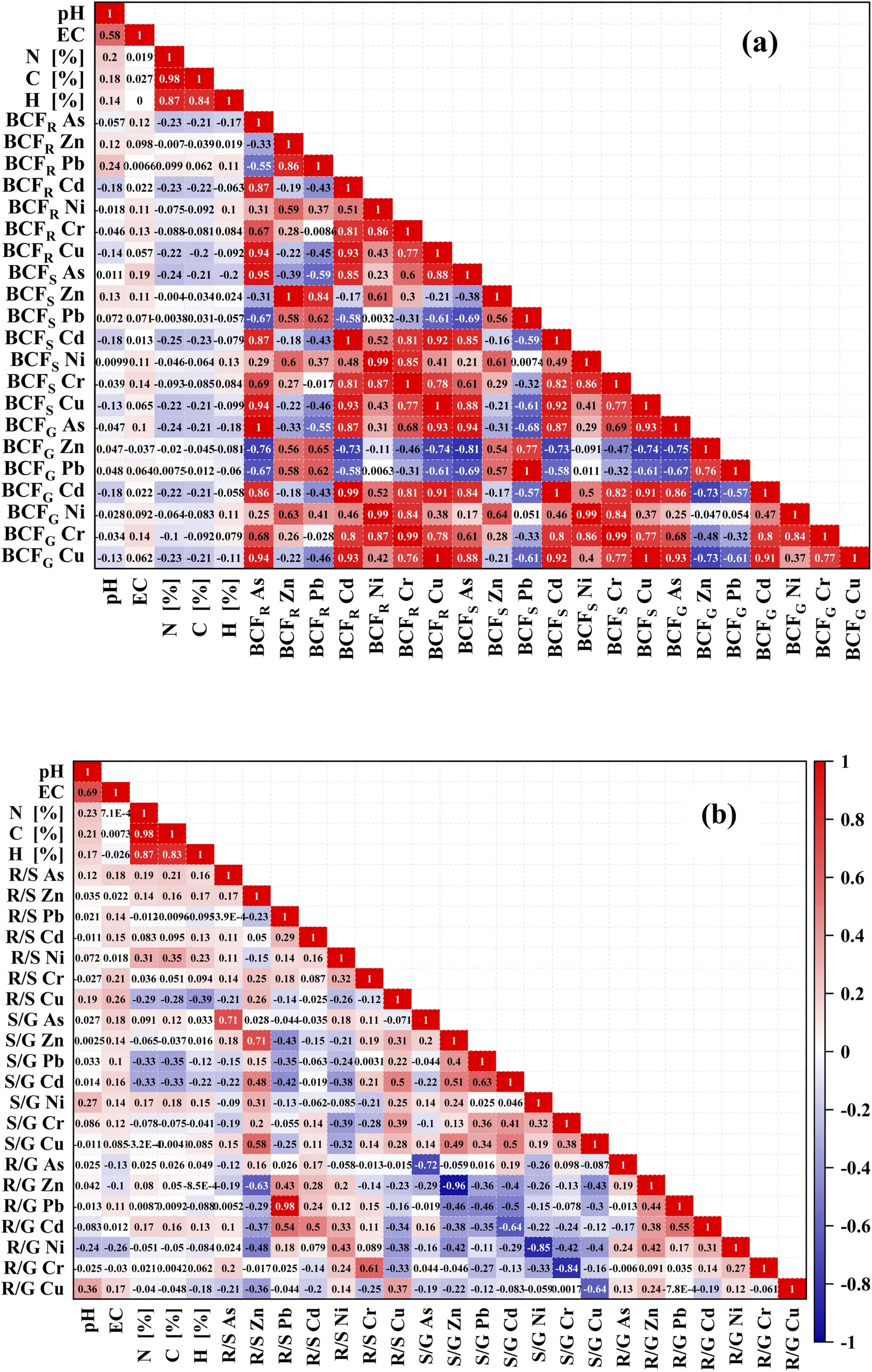
Correlation between soil physicochemical properties and rice metal (a) bioaccumulation and (b) translocation indices. BCF_R_, BCF_S_, and BCF_G_ denote bioaccumulation factors of metals in rice roots, shoots, and grains, respectively. R/S, S/G, and R/G indicate root-to-shoot, shoot-to-grain, and root-to-grain translocation factors. Magnitude of correlation is expressed as (*ρ*), blue (−1) represents strong correlation; red (+1) indicates strong positive correlation. Numbers within cells indicate the corresponding *ρ* values.

Relationships among soil physicochemical properties (pH, EC, N, C, H) and bioaccumulation factors of PTEs in rice tissues, including root (BCF_R_), shoot (BCF_S_), and grain (BCF_G_) (Fig. 10a) were determined using spearman correlation analysis. Correlation strength was classified as weak (|*ρ*| < 0.4), moderate (0.4–0.7), or strong (>0.7), and mechanistic interpretation was restricted to moderate-to-strong associations. Among the soil parameters, N and C showed a very strong positive correlation (*ρ* = 0.981, *p* < 0.01), while a moderate positive correlation was observed between soil pH and EC (*ρ* = 0.581, *p* < 0.01). Correlations between soil properties and PTE bioaccumulation factors were consistently weak (|*ρ*| < 0.25). For example, the relationship between soil N/C and BCF_R_ for As was weak and negative (*ρ* = −0.232). Because this association did not meet the predefined threshold for moderate correlation, it was not interpreted mechanistically. No PTE showed a moderate or strong correlation with any measured soil physicochemical properties, indicating that bulk soil chemistry (pH, EC, N, C, H) was not strongly associated with tissue-specific PTE bioaccumulation in this dataset.

In contrast, strong correlations were observed among tissue-specific BCFs of the same PTE. For several PTEs, BCF_R,_ BCF_S_ and BCF_G_ were positively correlated. This pattern was particularly evident for Cd, where correlations between tissue-specific BCFs were high (BCF_R_ Cd *vs.* BCF_S_ Cd: *ρ* = 0.998). Negative correlations were also observed between certain metals, including root As and Pb bioaccumulation (BCF_R_ As *vs.* BCF_R_ Pb: *ρ* = −0.550) and between grain Zn accumulation and root As uptake (BCF_G_ Zn *vs.* BCF_R_ As: *ρ* = −0.758).

### 3.10 Influence of soil properties on PTE translocation within rice

Correlation analysis was applied to evaluate relationships between soil physicochemical properties and internal translocation factors of PTEs in rice, including root-to-shoot (R/S), shoot-to-grain (S/G), and root-to-grain (R/G) pathways. The correlation matrix (Fig. 10b) showed a very strong positive correlation between soil N and C (*ρ* = 0.981, *p* < 0.01). A moderate positive correlation was observed between soil pH and EC (*ρ* = 0.692, *p* < 0.01). The relationships between soil properties (pH, EC, N, C, H) and TF were generally weak (|*ρ*| < 0.25). Soil pH and EC showed only weak associations with Cu translocation (pH *vs.* R/G TF, *ρ* = 0.35; EC *vs.* R/S TF, *ρ* = 0.26). These associations were therefore treated as weak trends rather than evidence of mechanistic control. Similarly, soil organic matter indicators (N and C) showed weak negative correlations with the shoot-to-grain translocation of Pb and Cd (|*ρ*| < 0.3) and were not interpreted mechanistically.

The strongest correlations were observed among the TFs themselves, indicating associations among internal PTE-transfer pathways. Pb showed a very strong positive correlation between R/S and R/G TFs (*ρ* = 0.98) while Zn displayed a distinct internal regulation pattern, characterized by a very strong negative correlation between S/G and R/G factors (*ρ* = −0.95). Additionally, Cd and Ni translocation factors showed several moderate to strong correlations with those of other elements.

### 3.11 Human health risk assessment

**Fig. 11:**
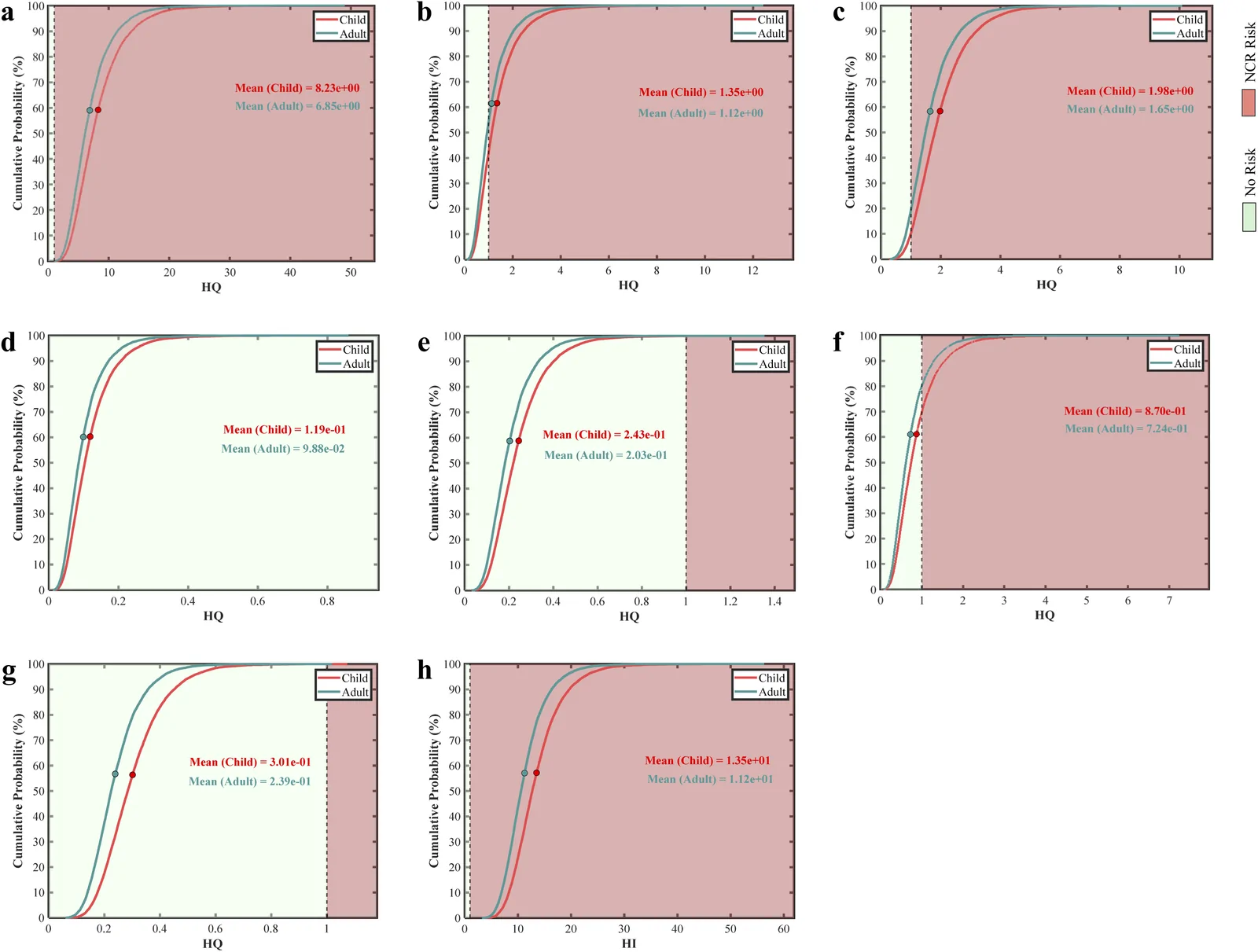
Cumulative probability distributions of non-carcinogenic hazard quotient (HQ) for potentially toxic elements in rice grains for children and adults: (a) As, (b) Cd, (c) Cr, (d) Cu, (e) Ni, (f) Pb, (g) Zn, and (h) total hazard index.

The cumulative probability distribution of HI values for rice ingestion in children and adults was used for probabilistic evaluation of non-carcinogenic risk (NCR) (Fig. 11). Among all analysed PTEs, As contributed the highest NCR with mean HI values of 8.23 for children and 6.85 for adults, exceeding the safety limit. The cumulative probability curves indicated that nearly the entire population, particularly children, experienced HI values greater than 1. Cd presented a significant concern, with mean HI values of 1.35 for children and 1.12 for adults, both exceeding the safety threshold, suggesting considerable non-carcinogenic risk through rice ingestion. Cr also demonstrated considerable risk for both exposure groups, with mean HI values of 1.98 for children and 1.65 for adults, indicating that a large proportion of the population is exposed to levels above the acceptable limit.

In contrast, Ni showed relatively lower NCR for both age groups, with mean HI values of 2.43 × 10^−1^ for children and 2.03 × 10^−1^ for adults. Pb exhibited moderate risk, with mean HI values of 8.70 × 10^−1^ for children and 7.24 × 10^−1^ for adults; although the average values remained below the safety limit, the upper tail of the child exposure distribution approached or exceeded HI = 1, indicating potential localized exposure risks. Similarly, Zn remained within acceptable risk levels, with mean HI values of 3.01 × 10^−1^ for children and 2.39 × 10^−1^ for adults. Cu showed the lowest contribution to non-carcinogenic risk, with mean HI values of 1.19 × 10^−1^ for children and 9.88 × 10^−2^ for adults, well below the safety threshold. Considering the cumulative hazard index (HI), the results indicate unacceptable combined risk, with mean HI values of 1.30 × 10^1^ for children and 1.12 × 10^1^ for adults, both substantially exceeding the safe limit of 1.

**Fig. 12:**
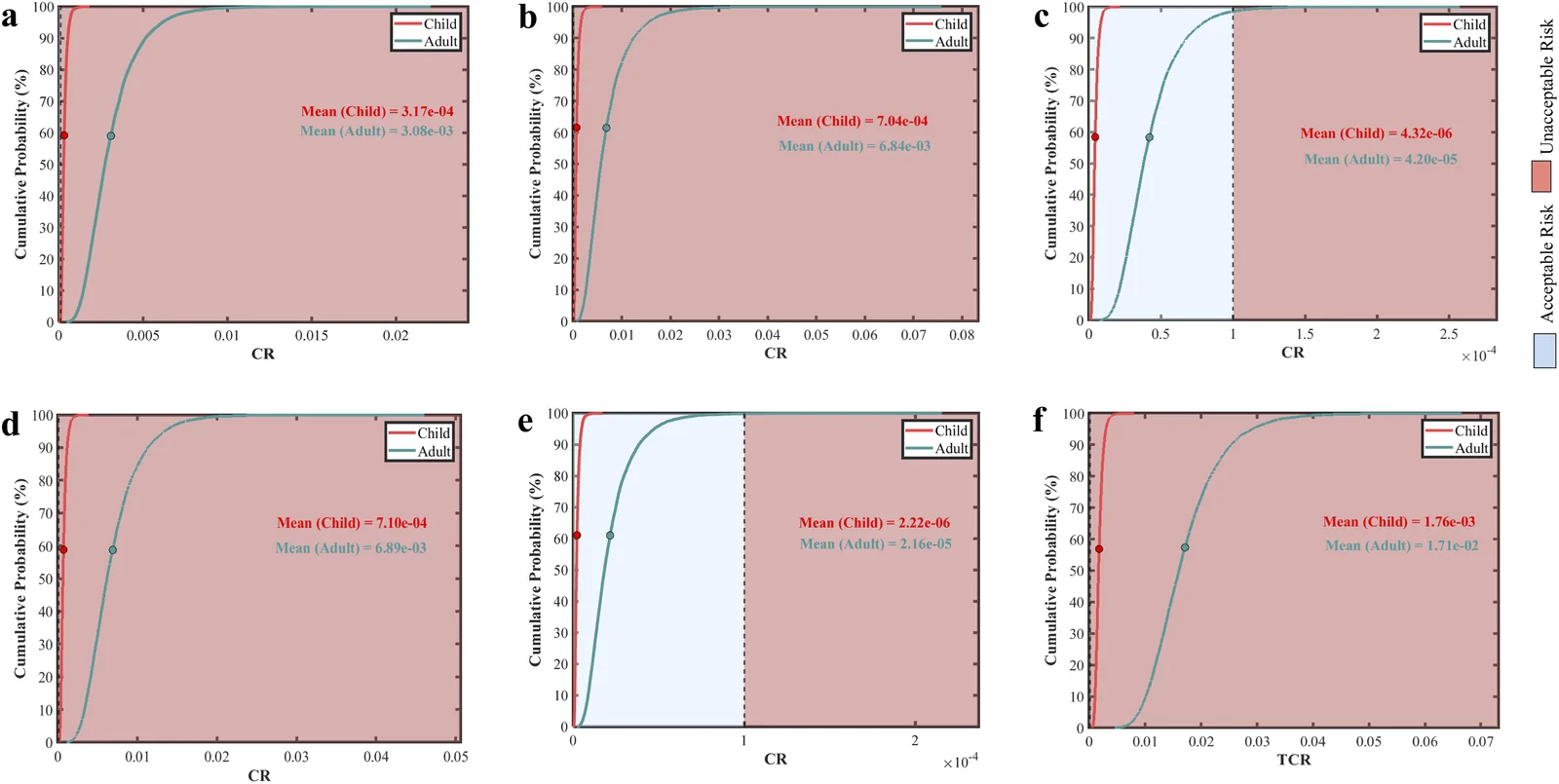
Cumulative probability distributions of carcinogenic risk (CR) for potentially toxic elements in rice grains for children and adults: (a) As, (b) Cd, (c) Cr, (d) Ni, (e) Pb, and (f) total cancer risk.

The cumulative distribution plots of CR illustrated As, Cd, and Ni as major contributors to carcinogenic risk (Fig. 12). The mean CR values for As were 3.17 × 10^−4^ for children and 3.08 × 10^−3^ for adults, both exceeding the acceptable risk threshold. The cumulative probability curves indicate that a large proportion of the population, particularly adults, experienced CR values above 1.00 × 10^−4^, confirming unacceptable carcinogenic risk from As exposure, with adults showing higher lifetime risk due to longer exposure duration.

The mean CR values for Cd were 7.04 × 10^−4^ and 6.84 × 10^−3^ for children and adults, respectively, were higher than the permissible value of 1.0 × 10^−4^. The risk distribution showed that almost entire population in the exposure scenario is within the unacceptable zone, suggesting that Cd is carcinogenic in nature and needs attention for controlling CR risk in the study area. Similarly, the CR associated with Cr was comparatively low. The mean CR values of 4.32 × 10^−6^ and 4.20 × 10^−5^ for children and adults were lower than the CR limit values. CR risk of Ni was high despite comparatively lower noncarcinogenic risk. The mean CR values of 7.10 × 10^−4^ and 6.89 × 10^−3^ for children and adults respectively. Low CR risk was found in the case of Pb with mean CR values of 2.22 × 10^−6^ and 2.16 × 10^−5^ for children and adults respectively.

**Fig. 13:**
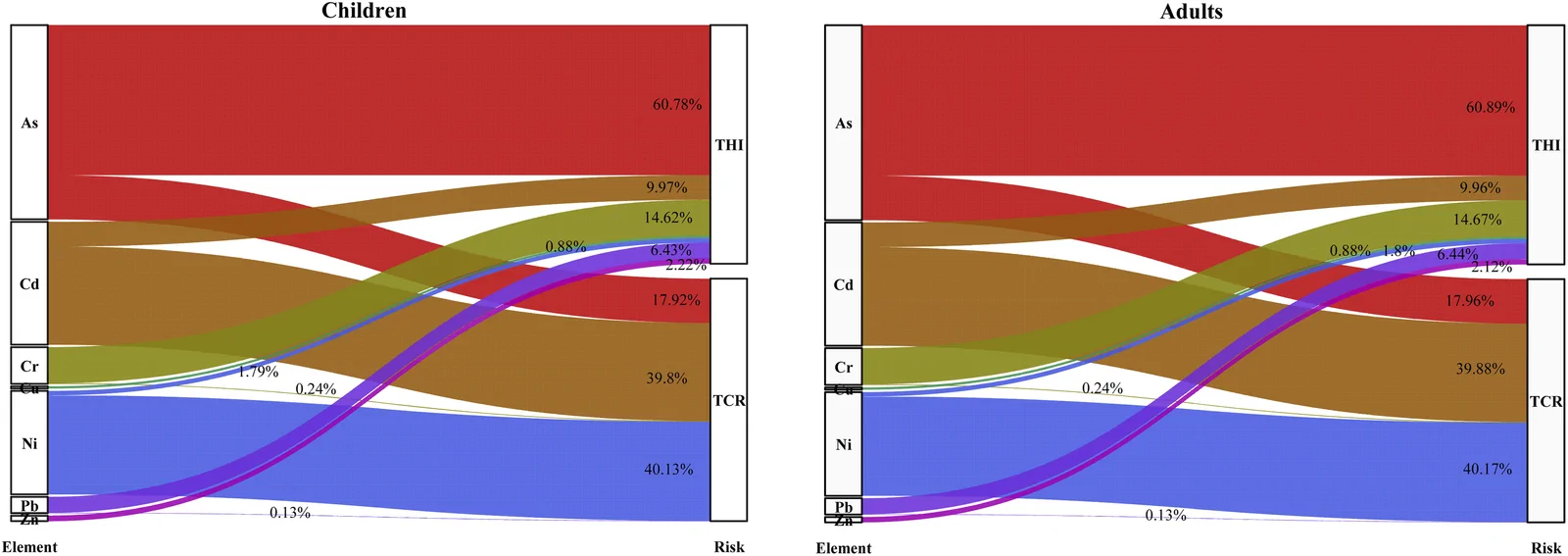
Relative contribution of individual PTEs to total non-carcinogenic risk (THI) and total carcinogenic risk (TCR) in rice grains for children and adults. Flow widths represent the proportional contribution (%) of each metal to the overall risk.

When considering the total carcinogenic risk (TCR), the results indicated unacceptable levels of risk for both age groups. Mean TCR values were 1.77 × 10^−3^ and 1.72 × 10^−2^ for children and adults respectively, higher than the benchmark value (1.00 × 10^−4^). As seen in risk distribution analysis, a significant portion of the exposed population was within the high-risk zone, confirming that As, Cd and Ni as major contributors to CR risk. As dominated non-carcinogenic risk (Fig. 13), comprising 60.7% of the aggregate HI, with Cr (14.6%) and Cd (∼9.9%), respectively, while Ni and Cd were the primary drivers of CR, accounting for over 80% of the total cancer risk, along with ∼17.9% for As.

### 3.12 Sensitivity analysis

**Fig. 14:**
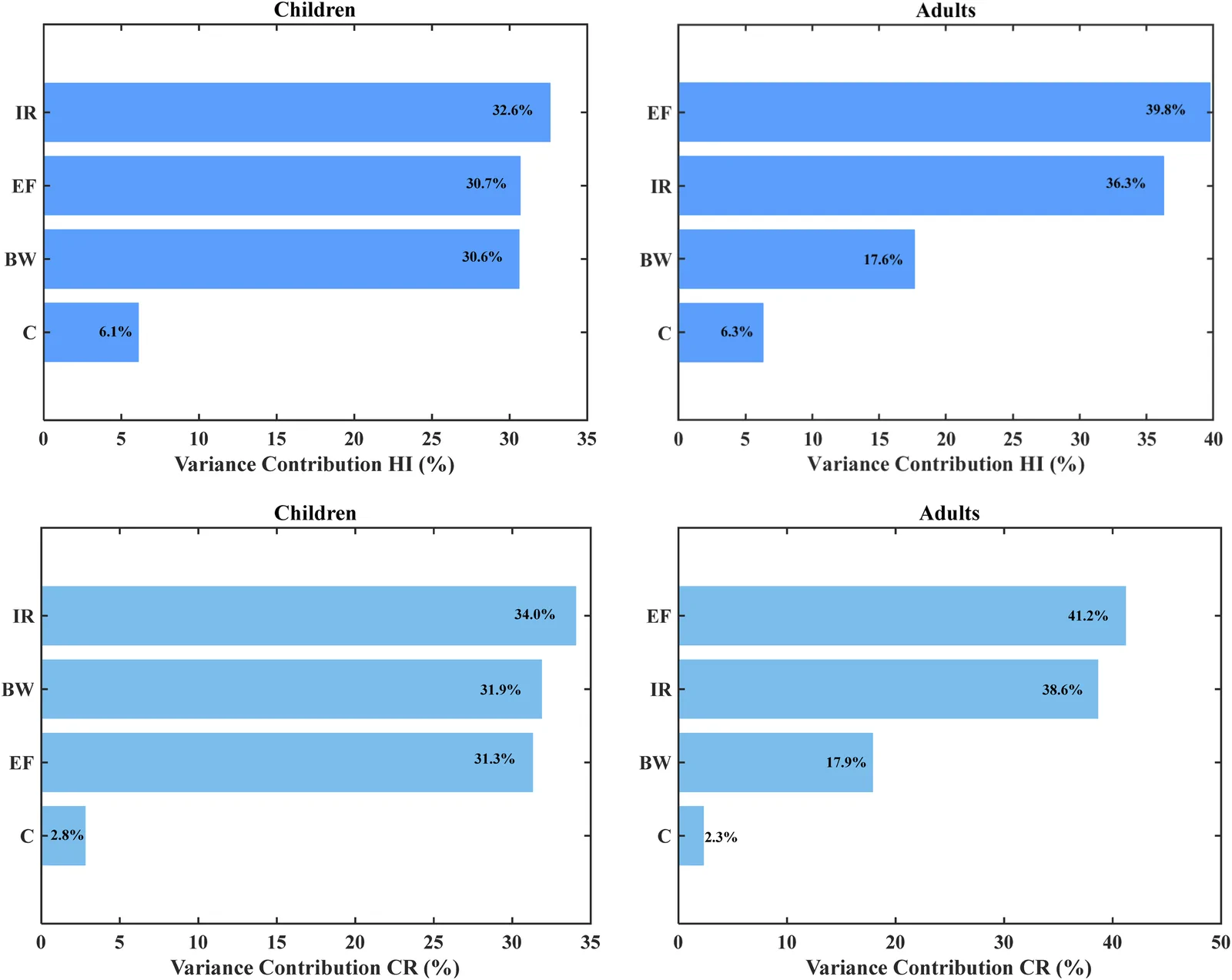
Sensitivity (variance contribution%) of exposure parameters influencing non-carcinogenic hazard index (HI) and carcinogenic risk (CR), C: PTE concentration, BW: body weight EF: exposure frequency IR: ingestion rate.

Uncertainty is an inherent feature of any model-based human health risk assessment, because of population-related differences in age, consumption patterns, body weight, and exposure conditions. In the current study, sensitivity analysis was carried out by Monte Carlo simulation to determine the contribution of major human exposure factors: metal concentration in rice, ingestion rate, exposure frequency, and body weight to the uncertainty in HI and CR for both children and adults (Fig. 14).

For NCR, sensitivity analysis suggested that IR and EF were the leading contributors for both children and adults, contributing to 32.6% and ∼30.7%, respectively, whereas the influence of BW was 30.6% with its negative inverse controlling effect on the risk. The influence of metal concentration was relatively smaller, accounting for ∼6% of the total variance, while for adults, the sum of EF and IR could explain more than 76% of the total variance in HI, with BW contributing to ∼17.6% and the influence of concentration being small (6.3%) as in the case of children, which shows that dietary behaviour and frequency of exposure are the major governing factors for the uncertainty in the non-carcinogenic risk.

Similarly, for CR, the patterns were comparable as IR, EF, and BW were the main contributing factors to total variance (34.0%, ∼31.3%, and ∼31.9%, respectively) in children, whereas the influence of concentration was small (2.8%). The dominant influence of EF and IR (41.2% and 38.6%, respectively) was seen in adults, with a minor contribution from BW (17.9%) to total variance, and again the impact of concentration uncertainty was negligible (2–3%) as in the case of children, which shows that long-term exposure and physiological parameters have a greater impact on carcinogenic risk than concentration variability. Even though BW had a negative effect on the risk, its significant contribution to uncertainty in total risk could be attributed to its strong controlling effect. In both cases, the sum of the contributions of IR, EF, and BW was significant, which shows that ingestion-related parameters play a major role in controlling health risk outcomes. Overall, the sensitivity analysis indicates that exposure-related behaviour and physiological characteristics exerted a greater influence on risk variability than concentration uncertainty, supporting the value of probabilistic assessment for characterizing dietary exposure risk.

## 4 Discussion

### 4.1 Spatial distribution of PTEs and industrial influence

As illustrated by Fig. 2, the spatial distribution of PTEs was characterized by heterogeneous contamination plumes extending away from the TPP and ash pond, confirming a point-source influence from the industrial facility. The observed spatial gradients are consistent with the combined influence of atmospheric deposition and lateral flow transport mechanisms.^46^ Fly ash carried by the winds settles on nearby rice production fields,^47,48^ after which, PTEs are laterally moved by surface runoff and irrigation return flows into the soils.^49^ Similar spatially heterogeneous and plume-like patterns have been reported in agricultural areas surrounding coal-fired thermal power plants.^50,51^

The plume distribution of PTEs is in line with the principal component pattern observed from PCA that showed that a large fraction of the variance (PC1) in the data is shared by the metals. This is consistent with the trace metal fingerprints of coal fly ash.^52,53^ The consistency between the source distribution pattern revealed by PCA and the spatial plume pattern of the contamination supports the hypothesis that these PTEs primarily originate from the coal-fired TPP. This contrasts with a hypothesis that the variation in concentrations could have been attributed to spatial differences in lithology or mineralogy, as such differences are typically associated with spatial patterns that are unrelated to the location of the TPP and ash pond.

The spatial patterns observed in the present study provide supporting evidence for an industrial influence, particularly around the TPP and ash pond, although the contribution of individual sources cannot be established solely from spatial interpolation or PCA. The broad enrichment patterns of Cr and Ni around the TPP and ash pond, together with the localized distributions of As, Cd, Cu, and Zn, suggest that PTE transport is influenced by both source proximity and local environmental conditions.^54,55^ Elevated Cd, Ni, and Cr in the principal enrichment zones are consistent with the occurrence of these elements in coal and coal combustion residues. In contrast, the less spatially coherent distributions of Cu and Zn suggest that additional sources may contribute to their occurrence, including agricultural inputs such as fertilizers and pesticides, as well as traffic and other local anthropogenic activities.^56^

The PCA results further indicated a strong common covariance among PTE concentrations across soil and rice tissues. However, because the PC1 loadings were relatively low and broadly similar across variables, PCA alone does not identified PC1 specifically as a TPP or fly-ash source. The interpretation of industrial influence is therefore strengthened by the spatial correspondence of elevated concentrations with the TPP and ash pond, together with the known occurrence of these PTEs in coal combustion residues, rather than by PCA alone.

The persistence of elevated PTE concentrations in agricultural soils, even where concentrations remained below selected regulatory guidelines, suggests that long-term and repeated low-level inputs may contribute to cumulative soil enrichment. This is consistent with the spatial gradients observed in Fig. 2 and with the role of agricultural soils as long-term sinks for atmospheric and surface-deposited contaminants. In addition to industrial inputs, repeated application of agricultural amendments and irrigation return flows may contribute to the retention and redistribution of PTEs within cultivated soils.^57^ Thus, the observed contamination pattern likely reflects the combined influence of point-source emissions and local agricultural and transport-related inputs, rather than a single pathway operating uniformly across the study area.

### 4.2 Soil pollution indices and legacy contamination

The observed enrichment patterns are consistent with the long-term accumulation of PTEs in agricultural soils influenced by industrial activities, particularly where repeated deposition of coal-combustion residues may occur. Coal combustion and ash handling can release PTE-bearing particulates that are subsequently deposited on surrounding soils, while runoff and irrigation can facilitate their redistribution within agricultural landscapes.^58,59^ The heterogeneous enrichment observed across the study area therefore likely reflects the combined effects of source proximity, atmospheric deposition, surface transport, and local soil-retention processes rather than a uniform contamination pathway. The pronounced enrichment of Cd is of environmental concern because Cd can remain relatively mobile and bioavailable in agricultural soils depending on pH, organic matter, mineral composition, and redox conditions.^60,61^ Its persistence in cultivated soils may facilitate continued transfer into crops even when total soil concentrations do not uniformly indicate severe contamination.^62^ This highlights the importance of considering both enrichment and subsequent plant uptake when evaluating the environmental significance of PTE contamination.

PLI provides an integrated measure of multi-element contamination, but it can mask the contribution of elements with greater toxicity or ecological significance.^63^ A moderate overall pollution status does not necessarily imply low environmental risk when individual elements such as Cd show disproportionate enrichment. Therefore, the combined interpretation of contamination indices with ecological risk and soil–plant transfer metrics provides a more meaningful assessment of the potential consequences of PTE accumulation in agricultural soils.^64^ Moreover, composite indicators such as PLI can obscure the impact of high-priority contaminants through averaging. By averaging metal concentrations, this index may underestimate the risks to crops grown in contaminated areas where bioavailability impacts plant uptake.^65^ Overall, the findings suggest that continued PTE inputs to agricultural soils surrounding coal-fired power infrastructure may contribute to a legacy contamination problem, with potential implications for long-term soil quality and crop safety.

### 4.3 Partitioning of PTEs between rice root and shoot

The distinct partitioning of PTEs between rice roots and shoots reflects the combined influence of soil geochemistry, rhizosphere processes and plant physiological regulation. In the present study, As, Pb and Cr showed greater retention in roots, whereas Cd and Ni exhibited comparatively greater mobility toward shoots.^66^ This element-specific behaviour indicates that total soil concentration is not the sole determinant of plant uptake and internal distribution.^67^ Instead, PTE speciation, sorption desorption reactions, root-surface adsorption and intracellular sequestration determine the fraction that becomes available for membrane uptake and subsequent translocation. Once absorbed, metals may be immobilized through binding to cell-wall functional groups, complexation with phytochelatins and metallothioneins, or vacuolar sequestration, thereby restricting their movement from roots to shoots.^68^ These patterns provide a mechanistic basis for the predominantly low root-to-shoot TF values observed in the present study and are consistent with the strong root retention of Pb, As and Cr.

The distinct variation in partitioning of different metals in rice roots and shoots is related to both soil and plant factors. Higher retention of As, Pb and Cr in roots compared to other metals is associated with specific plant mechanisms that sequester and immobilize metals in root tissue, such as by cell wall binding, interaction with phytochelatins and vacuolar sequestration.^2,69,70^ These mechanisms may help protect plants against toxic effects by limiting the translocation of metals from roots to shoot tissues.^71^ These processes may restrict movement from roots to shoots and provide a plausible explanation for the generally low root-to-shoot TF values observed in the present study. The pronounced retention of Pb in roots is particularly consistent with the strong binding of Pb to cell-wall components and its limited mobility within plant tissues.^72,73^ On the other hand, Cd and Ni show higher accumulation in the shoots compared to roots, associated with higher mobility and retention in shoot tissues.^74^ For instance, the movement of Cd is relatively effective in rice at low soil concentrations as it can be moved to the shoots by divalent cation transporters such as OsNRAMP5.^75^ Ni may similarly exhibit relatively high mobility because Ni^2+^ can be transported through pathways associated with divalent cations and may form complexes with organic ligands that facilitate movement through the transpiration stream.^76^ Zn showed relatively high concentrations in both roots and shoots, consistent with its essential role in plant metabolism and the homeostatic regulation of Zn uptake and transport through zinc-regulated/iron-regulated transporter-like proteins and related transporter families.^77,78^

Root-surface iron plaque formation provides an additional plausible physicochemical mechanism for the retention of As, Pb, and Cr in flooded rice systems.^79^ Under reducing conditions associated with submerged paddy soils, Fe released from soil minerals can move toward the oxygenated rhizosphere and precipitate as Fe (hydr)oxide coatings on root surfaces.^80^ These coatings provide reactive surfaces capable of adsorbing or co-precipitating PTEs, particularly As and Pb.^81^ Although iron plaque was not directly quantified in the present study, this mechanism provides a plausible explanation for the observed root retention and restricted root-to-shoot transfer of As, Pb, and Cr. Variation in PTE partitioning among sampling locations further indicates that local exposure conditions and rhizosphere processes influence plant uptake and distribution.^35,82,83^ Thus, PTE transfer within rice is likely governed by the interaction between external soil conditions and element-specific physiological mechanisms rather than by soil concentration alone.

### 4.4 PTE transfer to rice grains

The lower concentrations of most PTEs in grains compared with roots and shoots indicate comparatively restricted accumulation in the edible compartment. This pattern is consistent with the presence of physiological barriers associated with vascular transport, selective transporter expression, nodal sequestration, and regulation of xylem-to-phloem redistribution during grain filling.^84^ Consequently, several PTEs, including Pb and Cr, were retained preferentially in vegetative tissues, resulting in comparatively lower grain concentrations.^85–87^ In field experiments, it is often found that the concentration of grains accounts for only a small portion of the total concentration of a metal in the plant. This further highlights the significance of plant internal partitioning as a mechanism in safeguarding edible plants.^88–90^

However, the grain-transfer behaviour was strongly element-specific. As and Cd showed comparatively greater grain BCF values than several other PTEs, indicating that these elements may bypass some of the physiological barriers that restrict movement of other contaminants. As can enter rice through phosphate transport pathways because arsenate is a structural analogue of phosphate, whereas Cd can exploit transport systems associated with essential divalent cations.^91,92^

The efficient grain-directed translocation of As and Cd relative to other PTEs can be further understood through nutrient-competition and ionic-mimicry mechanisms operating at the point of uptake. Arsenate (As(v)), the predominant As species under oxidised conditions, is a structural analogue of inorganic phosphate and is consequently taken up *via* high-affinity phosphate transporters (OsPHT1 family) that are constitutively active in both vegetative and reproductive (grain-filling) tissues, allowing As to partially circumvent the compartmentalisation barriers that restrict most other PTEs to root and shoot tissue.^93^ Similarly, Cd^2+^ is not actively excluded by rice because it is transported as a non-essential mimic of essential divalent micronutrients (Zn^2+^, Mn^2+^, Fe^2+^) *via* broadly specific metal transporters, principally OsNRAMP5 and members of the ZIP family, which are expressed along the entire root-to-grain translocation pathway, including the nodal vascular bundles that govern preferential metal allocation to developing grains.^94,95^ These pathways provide a plausible physiological explanation for the relatively greater grain accumulation of Cd and As observed in the present study, although the relevant transporters and chemical species were not directly measured.

The wide variability in grain PTE concentrations further demonstrates that soil contamination alone cannot adequately predict dietary exposure. Cultivar characteristics, rhizosphere processes, PTE speciation, soil redox conditions, and internal translocation mechanisms may all influence grain accumulation. The absence of cultivar-specific information at individual sampling locations in the present study prevented direct evaluation of cultivar effects, highlighting the need for cultivar-resolved sampling in future investigations.

### 4.5 Influence of soil properties on PTE bioaccumulation in rice plant tissues

Generally weak and inconsistent correlations found between PTE BCFs in tissues of rice plant and physiochemical properties of the soil suggest that bulk soil properties alone did not explain the observed variability in tissue-specific PTE accumulation. N% and C% are positively correlated with each other since both share a strong relation to soil organic matter,^96^ while pH and EC showed a moderate positive relationship. Weak correlations of BCFs of As from soil and OM indicators (N and C) suggest low level reduction of metal bioavailability through sorption or complexation process,^97^ which might imply secondary role of soil organic matter in uptake of As rather than a direct control of metal uptake by the plant.^98^

Soil physicochemical conditions govern PTE bioavailability primarily through their control over metal speciation and solubility rather than through direct effects on plant physiology. Under the alkaline, moderately saline conditions characteristic of soils, Cd, Ni and Zn predominantly exist as more soluble, exchangeable or carbonate-bound fractions, whereas As, Pb and Cr tend to form less soluble Fe/Mn-oxide-associated or residual mineral fractions under oxidising conditions, consistent with the comparatively restricted root uptake.^98–101^ Upon field flooding for paddy cultivation, however, the shift to reducing conditions promotes reductive dissolution of Fe/Mn-oxides, which can transiently remobilise co-precipitated As and other oxyanion-forming metals into the soil solution, while simultaneously favouring sulphide formation that can immobilise Cd as insoluble CdS.^102,103^ This dynamic, redox-driven speciation, rather than the bulk soil chemical parameters measured here (pH, EC, N, C, H), likely accounts for the generally weak correlations observed between soil physicochemical properties and tissue-specific BCF values and underscores those uptake in flooded paddy systems is governed more by short-term redox-driven speciation than by static bulk soil fertility indicators.

Strong correlation between PTE BCFs in different parts of the plant highlights the significance of the plant specific mechanisms that controls uptake and translocation processes of PTEs in the whole system.^104^ Strong correlations of Cd BCFs in root, shoot and grain suggests a strong potential of soil-to-grain translocation of Cd due to Cd availability and its transport through divalent metal transporters.^105^ Negative correlations among some PTE-specific BCFs may also indicate interactions at uptake or transport sites.^106^ Thus, while soil properties can moderately impact availability of PTEs in soils, the findings imply that accumulation in different tissues of rice can be controlled by element specific uptake of PTEs under flooded paddy condition.

### 4.6 Influence of soil properties on PTE translocation within rice plant tissues

The relationships between soil physicochemical properties and TFs further indicated that external soil characteristics had relatively weak associations with PTE movement among rice tissues.^107^ Even though pH and EC control metal solubility and speciation in flooded paddy soils,^108^ most of their correlations with R/S and S/G were weak and metal dependent. This suggests that once PTEs are taken up by roots, their subsequent distribution is influenced more strongly by plant-internal regulatory processes than by the measured bulk soil properties.

A weak positive link between soil pH and Cu R/G indicates that Cu translocation might be facilitated by desorption and enhanced root uptake capacity in slightly basic soil.^109^ Meanwhile, EC showed a minor effect on Cu R/S transfer, possibly reflecting salinity-induced alteration of membrane permeability or ion competition between metals.^110,111^ On the other hand, weak but consistently negative correlations between indicators of soil organic matter and the Cd S/G and Pb S/G indicate that organic matter could potentially reduce accumulation of Cd and Pb in rice grains by promoting the fixation of metals in the soil or the reduction of mobility within the plant *via* complexation. Such behaviour is consistent with the findings in soil enriched with organic matter, where complexation and sequestration of metals restrict their mobility towards grains.^112,113^

The weak influence of soil properties suggests that PTE translocation is mainly controlled by plant-internal processes after root uptake. Xylem loading transfers PTEs from roots to shoots, while xylem unloading releases them into shoot tissues. HMA2/HMA3 regulate mainly Cd and Zn transport, whereas NRAMP transporters facilitate divalent-metal movement. Phloem remobilisation redistributes PTEs to grains during grain filling.^114,115^ The correlations among Cd^2+^, Ni^2+^, Zn^2+^ and Mn^2+^ suggest shared transport pathways and competition for transporter sites. Thus, grain accumulation depends not only on soil availability but also on competition among divalent cations within the plant.

The strong connections between the TFs themselves suggest that the movement of metals from roots and shoots to grain could follow the same routes. The tight link between the Pb R/S and R/G transfer factors suggests that variation in Pb movement to grains was closely associated with its movement from roots to shoots. Meanwhile, the strong negative association between Zn S/G and R/G indicates contrasting patterns of Zn redistribution among plant compartments.^107,116,117^ The associations between Cd and Ni transfer factors also suggest that these two metals may share the divalent metal transport system in controlling internal distribution.^118^ Overall, the weak relationships between soil properties and TFs, together with the stronger associations among tissue-specific TFs, indicate an important role for plant physiological regulation in determining PTE distribution within rice.

### 4.7 Indices of grain pollution and multi-metal exposure

The observed soil-to-rice transfer patterns indicate that PTE accumulation is controlled by a sequence of coupled processes rather than by soil concentration alone. These include PTE speciation and sorption in the soil, redox-driven changes in Fe/Mn mineral phases under flooded conditions, rhizosphere interactions and iron-plaque formation, adsorption and sequestration at the root surface, competition with essential nutrients for membrane transporters, and subsequent xylem and phloem redistribution within the plant.^119^ The relative contribution of each process is element-specific, which may explain why Pb and Cr showed stronger retention in below-ground tissues whereas Cd and Ni exhibited comparatively greater internal mobility.^120^ Importantly, these mechanisms provide a physiological basis for the observed discrepancy between soil contamination and grain contamination and demonstrate why food-chain risk assessment should incorporate bioaccumulation and translocation behaviour rather than relying exclusively on bulk soil pollution indices.

The GPF and MPI results further demonstrate that grain contamination represents a multi-element exposure problem rather than the exceedance of a single contaminant threshold.^121^ The relatively high proportion of sites with GPF ≥1 indicates widespread composite PTE loading in rice grains, while the element-specific MPI results revealed substantial differences among individual PTEs. Pb showed the greatest magnitude of exceedance, whereas Cr exhibited the highest frequency of sites exceeding its permissible limit, followed by Pb and Ni. These findings point to the potential discrepancy between soil contamination and grain safety for metals that differ in their translocation capacity and toxicological significance.^122,123^

Analogously, As, Cd and Cu were mostly within the safe ranges, however, they contributed positively to the overall pollution indices due to their accumulation effects, thereby highlighting the additive impact of multi-metal loadings.^124,125^ Even within the permissible range, these metals can contribute substantially to cancer risk given their well-documented carcinogenicity. Composite indices like MPI can therefore provide a holistic view of food quality by considering both additive and potentially synergistic impacts of metals, while GPF provides an overall measure of multi-element contamination.^126^ However, these indices should be interpreted alongside bioaccumulation, translocation, and probabilistic health-risk assessments because contamination indices alone do not account for differences in exposure behaviour, toxicity, body weight, or lifetime exposure. Hence, single-element safety thresholds alone may be insufficient in evaluating dietary metal risk in multi-contaminated agroecosystems. The assessment of integrated multi-element contamination is critical to improve the reliability of grain quality assessment and for the planning of appropriate risk management interventions in multi-polluted agricultural systems.^127^

### 4.8 Human health concerns of prolonged dietary consumption

Health risk assessment of chronic dietary consumption of PTEs through rice revealed a tendency of cumulative NCR and CR. Children exhibited greater non-carcinogenic risk, whereas adults showed higher lifetime carcinogenic risk under the adopted exposure assumptions, particularly because carcinogenic risk was averaged over a 70-year lifetime.^128,129^ Higher NCR was observed for As, whereas the predominant contributors to CR were As, Cd, and Ni.

Similar dietary risk patterns have been documented in other rice-consuming regions. A nationwide market-rice survey in Nepal reported a mean HI of 1.13 and mean TCR of 1.04 × 10^−3^, with As identified as the principal driver of non-carcinogenic risk and Cd of carcinogenic risk.^130^ This risk hierarchy is broadly consistent with the As-dominated non-carcinogenic and As/Cd/Ni-dominated carcinogenic risk observed in the present study, although the magnitude of risk was considerably higher here. In a soil–rice system in the Jin-Qu Basin, China, the hazard index for rice consumption was 2.141, with Cd and Pb identified as the main contributors to non-carcinogenic risk,^131^ broadly consistent with the elevated Cd- and Pb-related risk observed here despite differing source contexts. In the lower Brahmaputra Valley of northeast India, an agronomically comparable rice-growing system, non-carcinogenic risk was dominated by As (HQ > 1), while total cancer risk for adults and children ranged from 3.9 × 10^−3^ to 2.1 × 10^−2^, exceeding the acceptable threshold by up to two orders of magnitude, with variation observed across locally cultivated rice varieties including Mahsuri.^132^ In paddy systems in Malaysia, a study of metal partitioning in *Oryza sativa* tissues reported bioaccumulation factors below 1 for all metals, with the highest translocation occurring from soil to root and the lowest from leaf to grain,^133^ a partitioning pattern that closely corresponds to the root-dominant retention observed in the present study. Together, these studies reinforce that rice consumption can represent an important exposure pathway for PTEs even where soil contamination is not uniformly severe, particularly when specific PTEs exhibit efficient transfer to edible grain.

The elevated risks observed here are therefore better explained by the combined effects of grain concentrations, PTE toxicity, dietary intake, exposure frequency, and body weight, rather than uniformly high PTE uptake by rice. Despite generally low BCF and TF values, As, Cd, and Ni contributed substantially to carcinogenic risk, highlighting the limitations of relying solely on soil contamination to assess dietary exposure.^134^ These findings emphasize the need for regular monitoring of PTEs in rice grains, particularly around thermal power plants. Children warrant greater attention for non-carcinogenic risk, while lifetime carcinogenic risk should also be considered for adults, alongside cultivar-specific assessment and strategies to reduce PTE transfer to edible grains.

### 4.9 Study limitations and research recommendations

The study evaluated total PTE concentrations to provide a broad assessment of contamination and exposure levels. Although total PTE concentration is useful for the preliminary assessment of contamination, PTE speciation, which is important for accurately evaluating bioavailability and toxicity, was not considered. In addition, multivariate statistical approaches can provide insights into potential sources but cannot establish source contributions as conclusively as isotope-based approaches. Consequently, future studies should incorporate PTE speciation in rice grains to improve the characterization of bioavailable fractions and refine NCR and CR estimates. Furthermore, cultivar identity (Samba Mahsuri or MTU 1010) was not recorded at the individual sampling-site level during harvest, preventing assessment of cultivar-specific effects on PTE uptake. Future studies should therefore evaluate low-accumulating rice cultivars in combination with appropriate soil amendments and long-term monitoring as potential strategies for practical risk management.

## 5 Conclusions

This study presents a source-to-health risk framework linking coal-fired thermal power station emissions to soil contamination, rice bioaccumulation, and associated human health risks through dietary exposure. The findings suggest that agricultural soils under the direct influence of a coal-fired TPS act as a long-term repository of metals derived from combustion emissions, and rice grains act as a major and yet an unrecognized vector of exposure to PTEs under a moderate pollution status. The results also highlight the limitations of relying solely on soil pollution indices to evaluate food-safety risks, emphasizing the need for integrated, multi-compartment assessment of soil, plant tissues, and edible grains. The risk assessment results, particularly the elevated NCR for children, highlight the need for regular monitoring of PTE levels in staple food crops grown around coal-based thermal power plants. A combination of mitigation strategies should be implemented based on the identified transfer pathways. Soil amendments such as biochar, lime, and phosphate-based materials can be used to immobilise Cd and Pb. The use of rice cultivars with low Cd and Ni accumulation may also reduce PTE transfer to grains, given their high translocation potential. Alternate wetting and drying irrigation can help limit redox-driven As mobilisation in paddy soils. In addition, proper ash-pond containment and phytoremediation can help control contamination at its source. In the light of these findings, a future comprehensive investigation on metal speciation and source attribution using isotope analysis will provide valuable insights into the bio-accessibility, and source apportionment of PTEs, and contribute toward the development of more robust and targeted, risk-based regulatory frameworks for agroecosystems around industrial point sources of pollution.

## Author contributions

Likhitha Chekuri: investigation, formal analysis, writing – original draft. Zahid Bashir: investigation, methodology, data curation, formal analysis, visualization, software, writing – original draft. Deep Raj: conceptualization, validation, writing – review & editing, supervision.

## Conflicts of interest

There are no conflicts to declare.

## Abbreviations

ADD: Average Daily Dose
As: Arsenic
BCF: Bioconcentration Factor
BCF_G_: Bioconcentration Factor for Grain
BCF_R_: Bioconcentration Factor for Root
BCF_S_: Bioconcentration Factor for Shoot
BV: Background Value
BW: Body Weight
C: Carbon
CCME: Canadian Council of Ministers of the Environment
Cd: Cadmium
CF: Contamination Factor
CHNS: Carbon, Hydrogen, Nitrogen, Sulfur (elemental analyzer)
Co: Cobalt
CR: Carcinogenic Risk
CRM: Certified Reference Material
Cu: Copper
CV: Coefficient of Variation
EC: Electrical Conductivity
EF: Exposure Frequency/Ecological Risk Factor
EIA: Environmental Impact Assessment
ER: Ecological Risk factor
FSSAI: Food Safety and Standards Authority of India
GPF: Grain Pollution Factor
H: Hydrogen
HI: Hazard Index
HQ: Hazard Quotient
HRA: Human Health Risk Assessment
ICP-OES: Inductively Coupled Plasma Optical Emission Spectrometry
IDW: Inverse Distance Weighted
ILS: Indian Limit of Soil
IR: Ingestion Rate
MAC: Maximum Allowable Concentration
MPI: Metal Pollution Index
N: Nitrogen
NCR: Non-Carcinogenic Risk
Ni: Nickel
Pb: Lead
PC: Principal Component
PCA: Principal Component Analysis
PERI: Potential Ecological Risk Index
PLI: Pollution Load Index
PTEs: Potentially Toxic Elements
QA/QC: Quality Assurance/Quality Control
RfD: Reference Dose
R/G: Root-to-Grain
R/S: Root-to-Shoot
SD: Standard Deviation
SF: Slope Factor
S/G: Shoot-to-Grain
TCR: Total Carcinogenic Risk
TF: Translocation Factor
TPP: Thermal Power Plant
USEPA: United States Environmental Protection Agency
WHO: World Health Organization
Zn: Zinc

## Supplementary Material

RA-OLF-D6RA06383G-s001

## Data Availability

Data supporting the conclusions of this study are provided in the supplementary information (SI) file. Additional data are available upon reasonable request from the corresponding author. Supplementary information is available. See DOI: https://doi.org/10.1039/d6ra06383g.
