# Supplementary material for: Deciphering the soil–rice continuum: transfer dynamics and dietary risks of potentially toxic elements in paddy fields near a coal-fired thermal power plant
Source: RSC Adv. 2026 Sep 9;16(43):53640–65. doi: 10.1039/d6ra06383g (PMC13555700; doi:10.1039/d6ra06383g)
Supplement: Supplementary file 1 [file RA-016-D6RA06383G-s001.pdf]

Supplementary Information (SI) for RSC Advances.
This journal is © The Royal Society of Chemistry 2026

Supplementary Information (SI)

Deciphering the soil–rice continuum: Transfer dynamics and dietary risks of potentially toxic elements in paddy fields near a coal-fired thermal power plant

Table S1. The equations and classification of I_{geo}, *PLI, EF, CF* (Gong et al., 2024; Hakanson et al.1980, Tomlinson et al., 1980, Huang et al., 2021, Müller et al.,1969).

Index	Equation	Category	Degree
Contamination factor (*CF*)	$CF = \frac{C_n}{S_n}$	CF < 1	Non-pollution
		1 ≤ CF < 2	Low pollution
		2 ≤ CF < 3	Moderate pollution
		3 ≤ CF < 5	High pollution
		CF ≥ 5	Extremely high pollution
Pollution load index (*PLI*)	$PLI = \sqrt[n]{CF_1 \times CF_2 \times \cdots \times CF_n}$	0 < PLI < 1	Unpolluted
		1 < PLI < 2	Moderately polluted to unpolluted
		2 < PLI < 3	Moderately polluted
		3 < PLI < 4	Moderately to highly polluted
		4 < PLI < 5	Highly polluted
		5 < PLI	Very highly polluted
Ecological risk factor (EF)	$EF = T_r^i \times CF$	EF < 40	Low
		40 ≤ EF< 80	Moderate
		80 ≤ EF < 160	Considerable
		160 ≤ EF < 320	High
		≥320	Very high
Potential Ecological Risk Index (PERI)	$PERI = \sum_{i=1}^{n} EF$	<150	Low
		150–300	Moderate
		300–600	Considerable

		≥600	High

Where, Cn is the concentration of PTE 'n' in the soil, and Sn is the Indian natural background value of the PTE 'n' in soil (mg/kg). T_r^i is the biological toxicity factor, T_r^i values differ for each metal: 30 for Cd, 2 for Cr, 10 for As, 1 for Zn, 5 for Cu, 5 for Pb and 5 for Ni and Co (Huang et al., 2018).

Table S2. Uncertainty concentrations (mg/kg) of PTEs

PTE	Probabilistic distribution	Parameters (mean, SD)	Reference
As	Log normal	0.42, 0.21	This study
Zn	Log normal	34.25, 11.92	This study
Pb	Log normal	0.52. 0.32	This study
Cd	Log normal	0.23, 0.15	This study
Ni	Log normal	0.83, 0.40	This study
Cr	Log normal	1.01. 0.46	This study
Cu	Log normal	0.81. 0.36	This study

(MATLAB (2025b) was used to fit the probabilistic distribution of the uncertain concentrations of PTEs)

Table S3. Parameters and their detailed information for the health risk assessment (USEPA, 1992)

Equation	Parameters	Information	
$ADI_{ing} = \dfrac{C_n \times IR_{ing} \times EF \times ED \times CF}{BW \times AT}$	IR_{ing}	Ingestion rate	
	EF	Exposure frequency	
	ED	Exposure duration	
	BW	Average body weight	
	AT	Average time	Non-carcinogenic
			Carcinogenic
	CF	Unit conversion factor	

Table S4. Parameters for the exposure risk calculations of PTEs from rice ingestion with Mote Carlo simulation.

Description	Parameter	Unit	Type	Children	Adult	Reference
Exposure frequency	*EF*	day/year	Triangular	TRI (180, 345, 365)		(Huang et al., 2021)
Ingestion rate	IR_{ing}	kg/day	Triangular	0.236	0.362	(Yang et al., 2019)
Average body weight	*BW*	kg	Point	32.5	60	(Han et al., 2020)
Average time of exposure to contaminants	*AT*	day	Point	$365 \times ED$（non-carcinogenic）<br>365×70 (carcinogenic）		(Han et al., 2020)
Exposure durations	*ED*	years	Point	6	70	(Bashir et al., 2026)

Table S5. The reference doses (*RfD*) and slope factors (*SF*). (*RfD* for noncarcinogenic PTEs and SF for carcinogenic PTEs).

PTE	*RfD* (mg/kg·d)	SF (kg/d·mg)
	RfD_{ing}	SF_{ing}
As	3.00E-04[a]	1.50E+00[a]
Cd	1.00E-03[a]	6.10E+00[a]
Cu	4.00E-02[a]	N/A
Ni	2.00E-02[a]	1.70E+00[b]
Pb	3.50E-03[a]	8.50E-03[a]
Zn	3.00E-01[a]	N/A
Cr	3.00E-03[b]	8.50E-03[c]

N/A, Data not available,[a](Huang et al., 2021), [e](Tong et al., 2020), [c](Zhou et al.,2022),

Table S6. The classification of *HI* and *TCR* (*CR*) (UEPA, 1992)

Category	Degree
HI <1	No risk of adverse health effects
HI ≥1	The occurrence of non-carcinogenic effects.
CR ≤ 1.00E-06	A mild risk
1.00E-06 ≤ (*CR*) ≤ 1.00E-04	Acceptable risk
TCR (*CR*) ≥ 1.00E-04	Unacceptable risk

Table S7. Statistical summary of the Metal Pollution Index (MPI) for heavy metals in rice grains against FSSAI standards.

Metal	FSSAI Standard (mg kg⁻¹)	MPI Range (Min – Max)	Mean MPI (±SD)	Sites Exceeding Standard (MPI ≥ 1)
**Pb**	0.2	0.05 – 5.70	2.31 ± 1.60	31 (66%)
**Cr**	1.0	0.11 – 1.53	1.02 ± 0.54	34 (72%)
**Ni**	1.0	0.16 – 1.23	0.86 ± 0.34	31 (66%)
**Zn**	50	0.23 – 1.09	0.70 ± 0.25	16 (34%)
**Cd**	0.4	0.00 – 0.98	0.59 ± 0.37	0 (0%)
**As**	1.1	0.05 – 0.58	0.39 ± 0.18	0 (0%)
**Cu**	30	0.003 – 0.042	0.03 ± 0.01	0 (0%)

Table S8. Statistics and classification of the Grain Pollution Factor for rice grains.

Statistic	Value
**Range**	0.11 – 1.64
**Mean (±SD)**	1.03 ± 0.46
**Sites with GPF < 1 (Low Pollution)**	13 (27.7%)
**Sites with 1 ≤ GPF< 3 (Moderate Pollution)**	34 (72.3%)
**Sites with GPF > 3 (Severe Pollution)**	0 (0%)

Table S9. Summary of Contamination Factor (CF) and Pollution Load Index (PLI) for soil heavy metals.

Parameter	As	Zn	Pb	Cd	Ni	Cr	Cu	PLI
CF Range	0.18 – 1.23	0.39 – 3.49	0.04 – 0.36	0.5 – 7.5	0.66–5.79	0.21 – 1.80	0.38 – 2.48	0.25–2.23
Mean CF (±SD)	0.81 ± 0.34	2.39 ± 0.96	0.24 ± 0.11	4.63 ± 1.97	3.82 ± 1.73	1.18 ± 0.54	1.73 ± 0.65	1.48 ± 0.65
Pollution Category	Non to Low	Low to Moderate	Non to Low	High to Extremely High	Non-pollution to Extremely High	Non-pollution to Low	Non-pollution to Moderate	Unpolluted to Moderately/Heavily Polluted

*Note: CF Categories: CF < 1 (Non-pollution); 1 ≤ CF < 2 (Low); 2 ≤ CF < 3 (Moderate); 3 ≤ CF < 5 (High); CF ≥ 5 (Extremely High). PLI Categories: 0 < PLI < 1 (Unpolluted); 1 < PLI < 2 (Moderately polluted to unpolluted).

Table S10. Statistical summary of the Ecological Risk Factor (EF) and Potential Ecological Risk Index (PERI) for soil heavy metals.

Parameter	Range	Mean	Risk Category (All Sites)
ER for Cd	2.5–37.5	23.88	Low (ER < 40)
ER for Ni	19.71–173.61	114.50	Considerable (ER = 80–160)
ER for As	1.78–12.29	7.87	Low (ER < 40)
ER for Zn	0.39–3.49	2.26	Low (ER < 40)
ER for Cu	0.76–4.96	3.47	Low (ER < 40)
ER for Cr	1.04–8.99	5.91	Low (ER < 40)
ER for Pb	0.19–1.83	1.19	Low (ER < 40)

Parameter	Range	Mean	Risk Category (All Sites)
PERI	26.37–241.41	159.08	Moderate (PERI = 150–300)

*Note: ER < 40 = Low; 40 ≤ ER < 80 = Moderate; 80 ≤ ER < 160 = Considerable; ER ≥ 160 = Very high.
PERI < 150 = Low; 150 ≤ PERI < 300 = Moderate; 300 ≤ PERI < 600 = Considerable; PERI ≥ 600 = Very high.

Table S11. Eigenvalues and variance explained by the principal components.

Principal Component	Eigenvalue	Variance Explained (%)	Cumulative Variance (%)
PC1	24.11	89.30	89.30
PC2	2.17	8.03	97.33
PC3	0.42	1.56	98.89
PC4	0.20	0.73	99.62
PC5-PC27	<0.05	<0.18	100.00

Table S12. Loadings of heavy metal variables on the first two principal components.

Component	Variable	Loading	Interpretation
PC1	All measured variables	~0.20	Below interpretive threshold; weak/negligible individual contribution.
PC2	Soil Pb	0.054	Negligible; below interpretive threshold

Component	Variable	Loading	Interpretation
PC2	Shoot Cd	−0.042	Negligible; below interpretive threshold
PC2	Root Cd	−0.039	Negligible; below interpretive threshold
PC2	Grain Cd	−0.037	Negligible; below interpretive threshold
PC2	Shoot Pb	0.671	Moderate-to-strong positive loading
PC2	Grain Pb	0.671	Moderate-to-strong positive loading

Table S13. Abbreviations and corresponding full forms of labels used in the PCA biplot.

**Abbreviation**	**Full form**
S-As	Soil As
R-As	Root As
Sh-As	Shoot As
G-As	Grain As
S-Zn	Soil Zn
R-Zn	Root Zn
Sh-Zn	Shoot Zn
G-Zn	Grain Zn
S-Pb	Soil Pb

**Abbreviation**	**Full form**
R-Pb	Root Pb
Sh-Pb	Shoot Pb
G-Pb	Grain Pb
S-Cd	Soil Cd
R-Cd	Root Cd
Sh-Cd	Shoot Cd
G-Cd	Grain Cd
S-Ni	Soil Ni
R-Ni	Root Ni
Sh-Ni	Shoot Ni
G-Ni	Grain Ni
S-Cr	Soil Cr
R-Cr	Root Cr
Sh-Cr	Shoot Cr
G-Cr	Grain Cr
S-Cu	Soil Cu
R-Cu	Root Cu

Abbreviation	Full form
Sh-Cu	Shoot Cu
G-Cu	Grain Cu

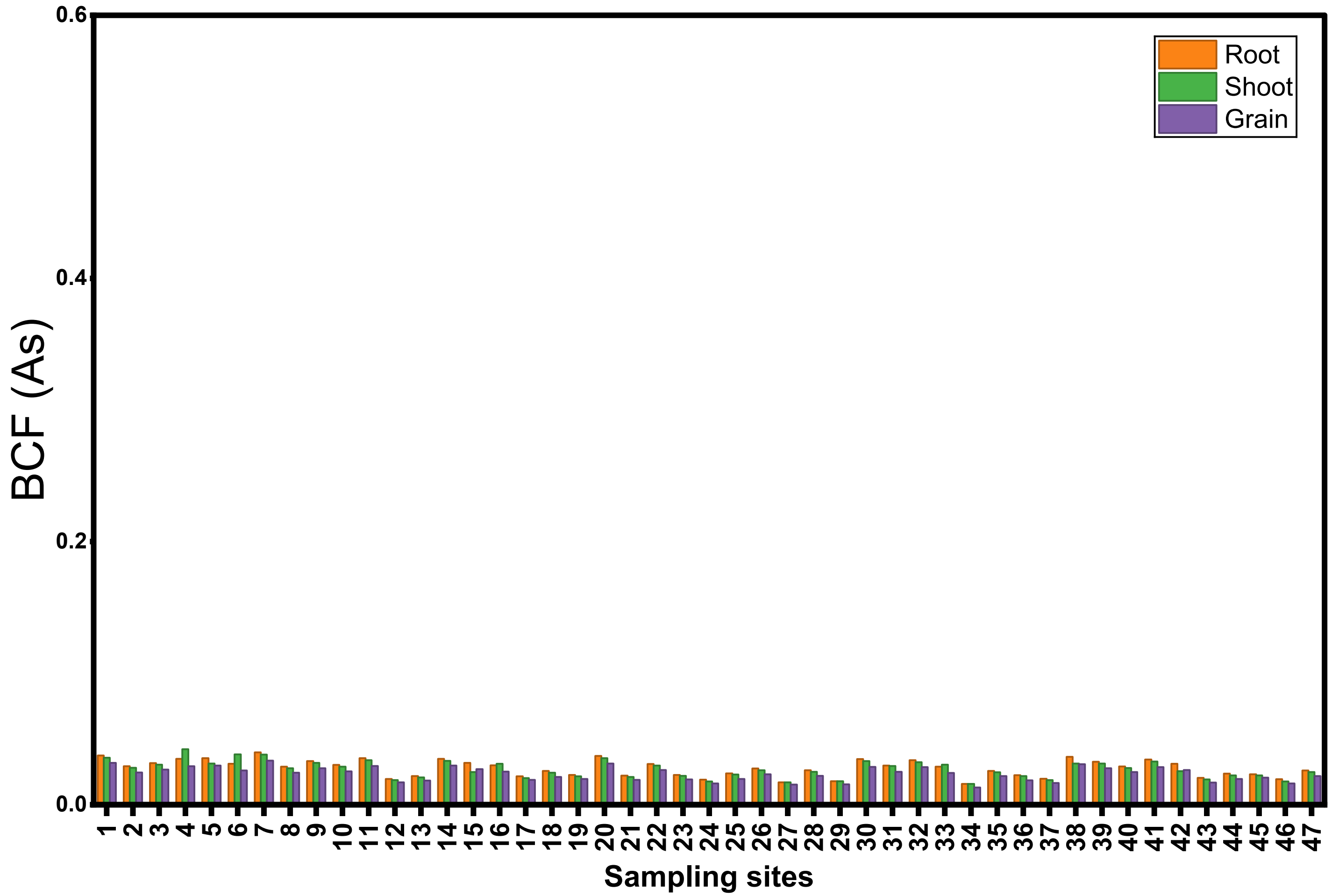
BCF (As)
0.6
0.4
0.2
0.0
Root
Shoot
Grain
1 2 3 4 5 6 7 8 9 10 11 12 13 14 15 16 17 18 19 20 21 22 23 24 25 26 27 28 29 30 31 32 33 34 35 36 37 38 39 40 41 42 43 44 45 46 47
Sampling sites

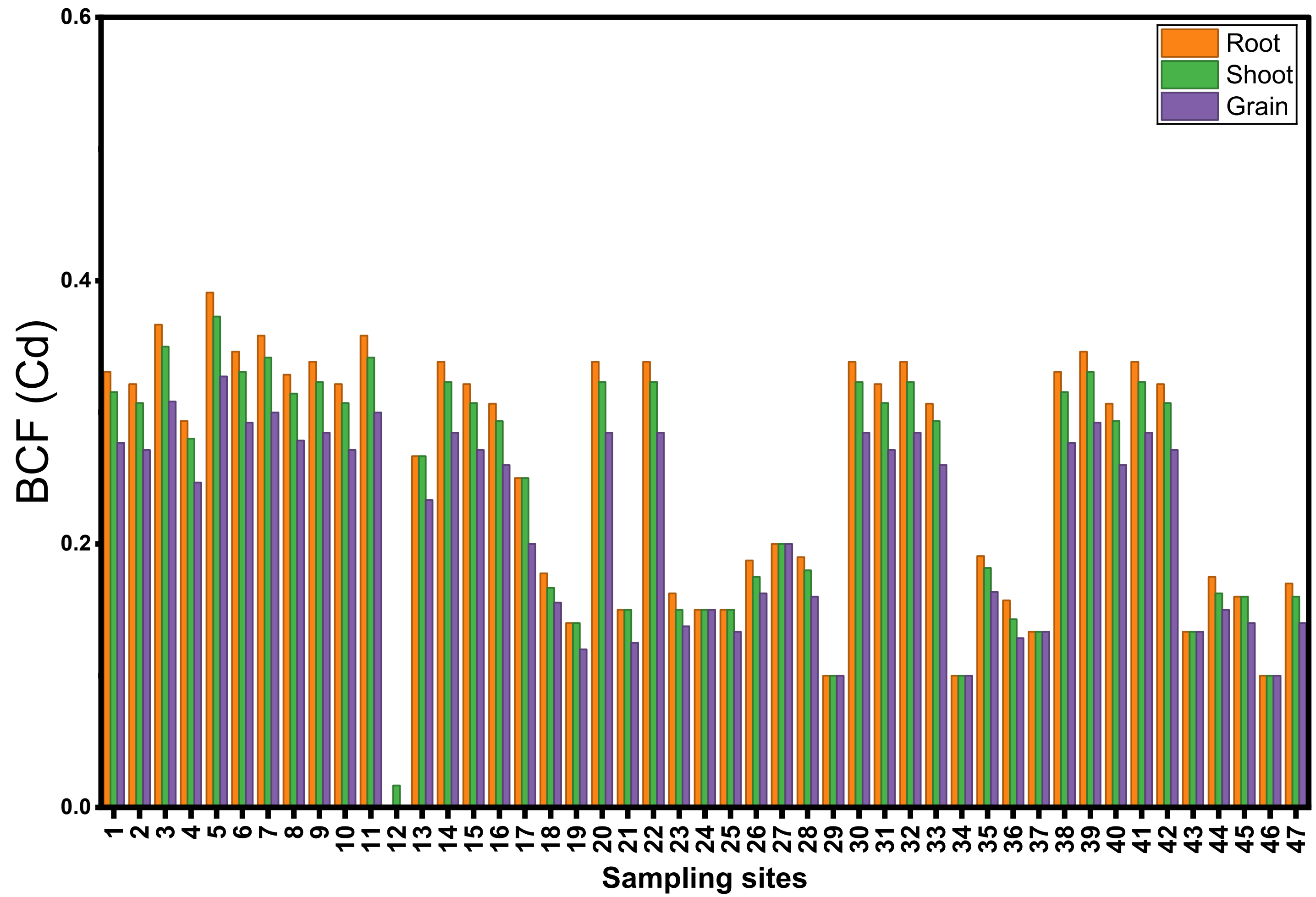
BCF (Cd)
0.0
0.2
0.4
0.6
Root
Shoot
Grain
1 2 3 4 5 6 7 8 9 10 11 12 13 14 15 16 17 18 19 20 21 22 23 24 25 26 27 28 29 30 31 32 33 34 35 36 37 38 39 40 41 42 43 44 45 46 47
Sampling sites

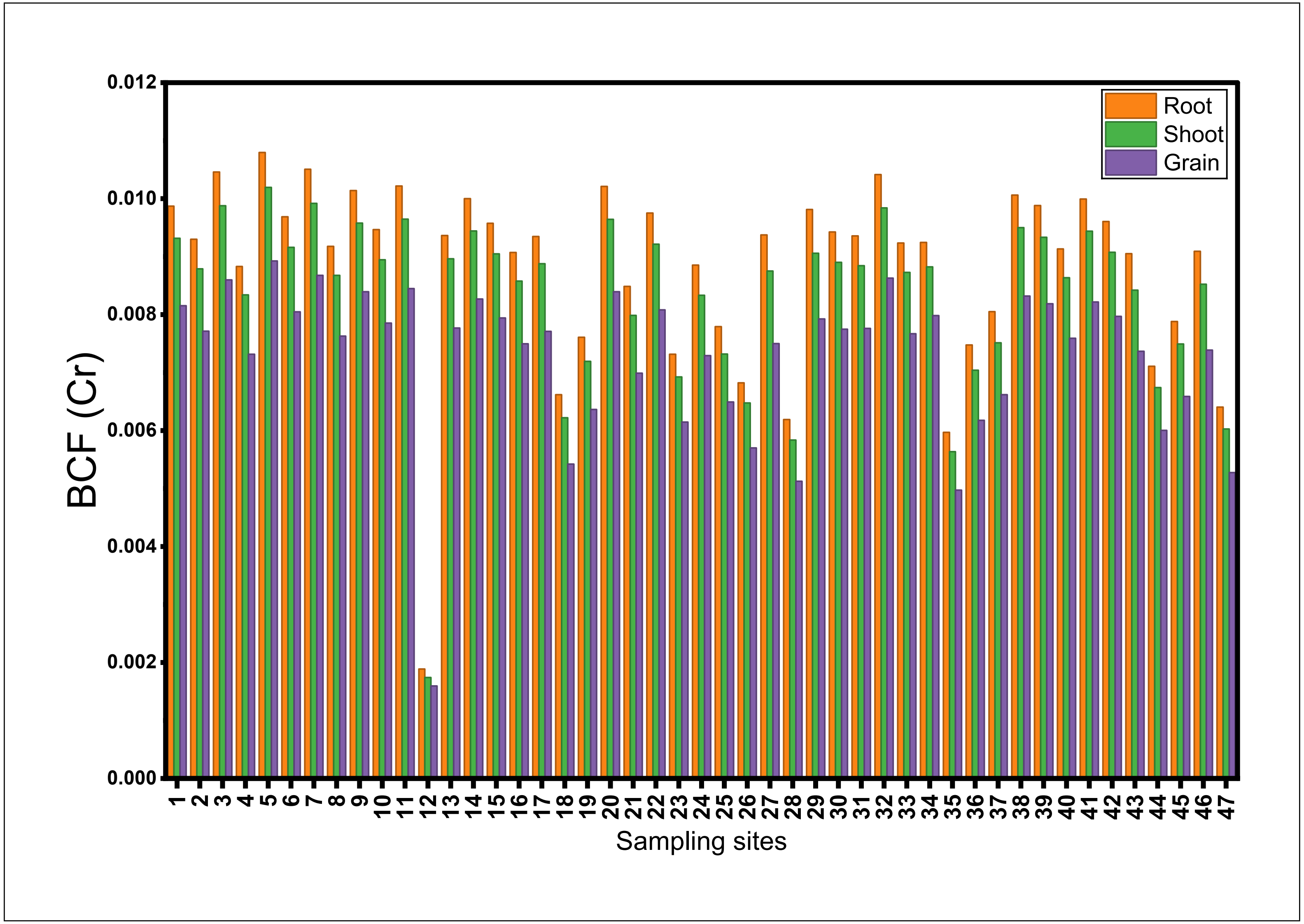

BCF (Cr)
0.012
0.010
0.008
0.006
0.004
0.002
0.000
Root
Shoot
Grain
1 2 3 4 5 6 7 8 9 10 11 12 13 14 15 16 17 18 19 20 21 22 23 24 25 26 27 28 29 30 31 32 33 34 35 36 37 38 39 40 41 42 43 44 45 46 47
Sampling sites

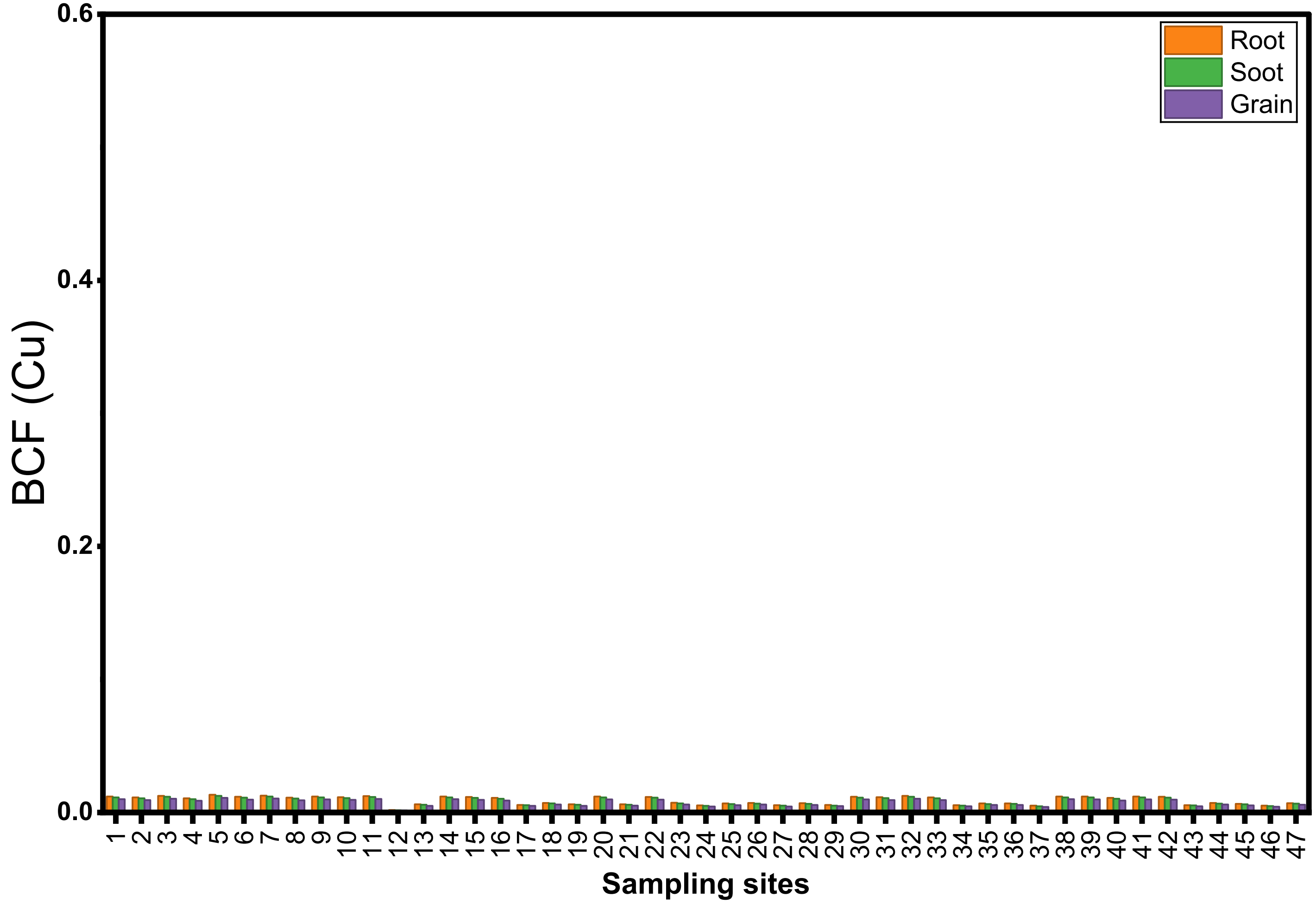
BCF (Cu)
0.6
0.4
0.2
0.0
Root
Soot
Grain
1 2 3 4 5 6 7 8 9 10 11 12 13 14 15 16 17 18 19 20 21 22 23 24 25 26 27 28 29 30 31 32 33 34 35 36 37 38 39 40 41 42 43 44 45 46 47
Sampling sites

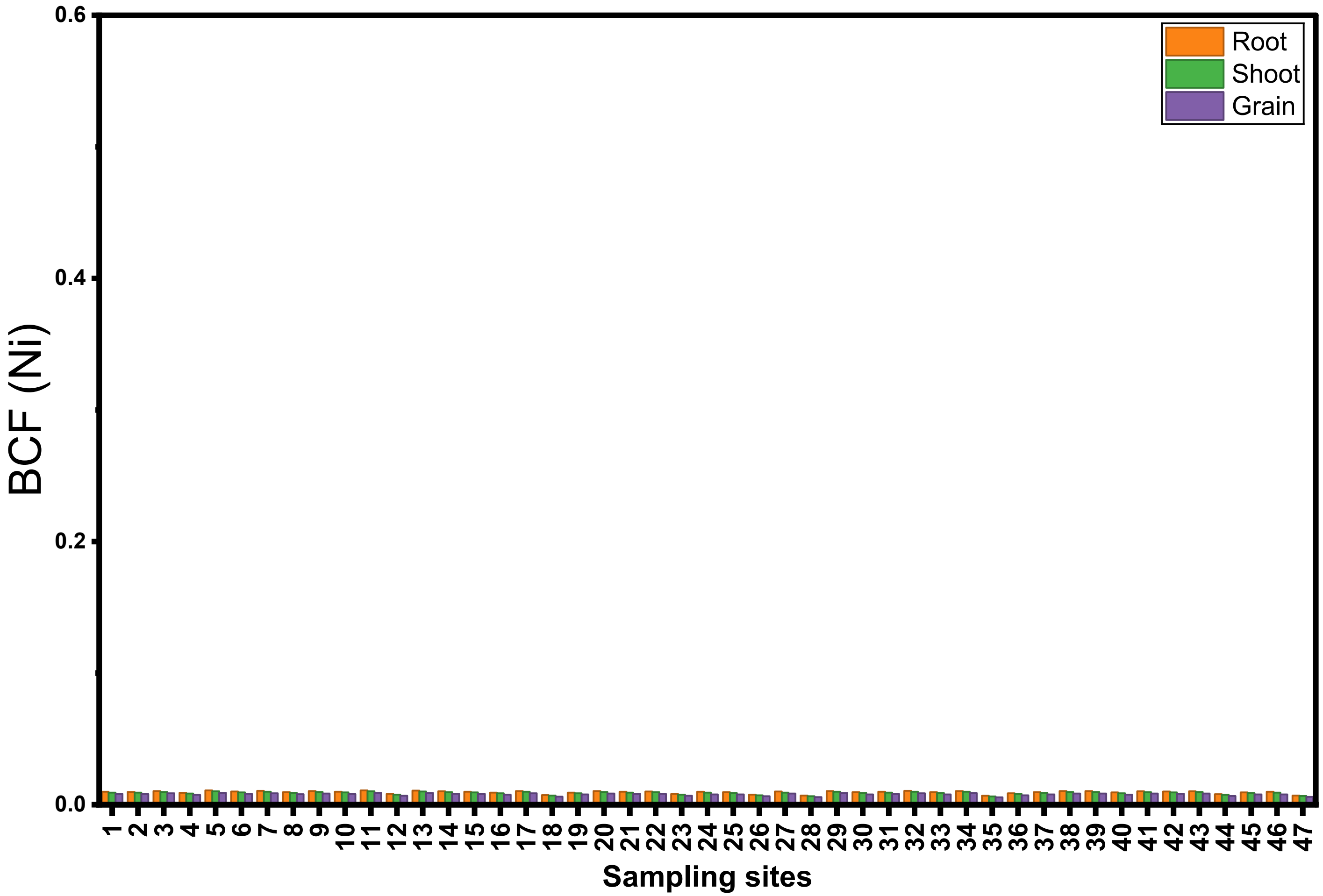

BCF (Ni)
0.0
0.2
0.4
0.6
Root
Shoot
Grain
1 2 3 4 5 6 7 8 9 10 11 12 13 14 15 16 17 18 19 20 21 22 23 24 25 26 27 28 29 30 31 32 33 34 35 36 37 38 39 40 41 42 43 44 45 46 47
Sampling sites

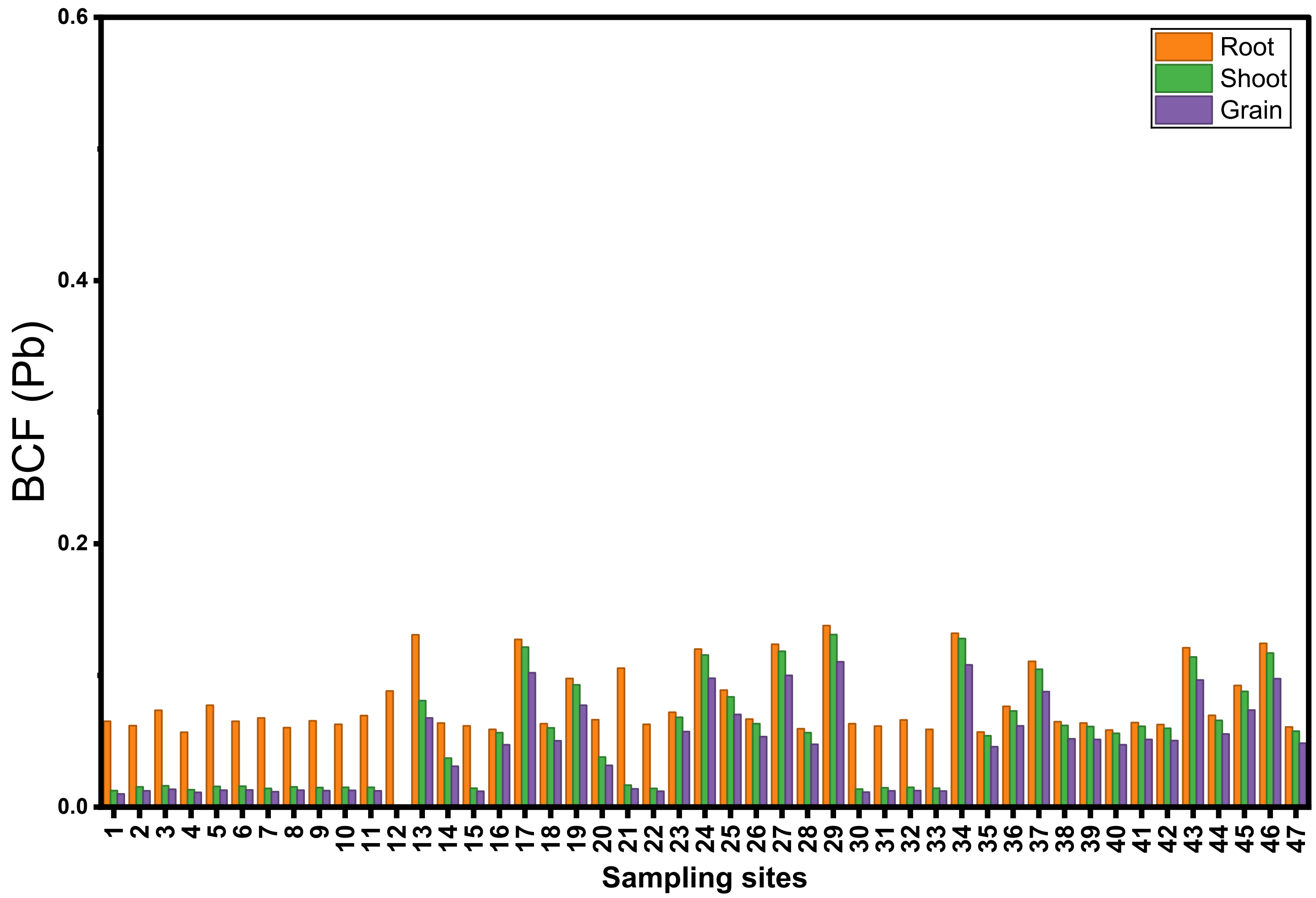

BCF (Pb)
0.6
0.4
0.2
0.0
Root
Shoot
Grain
1 2 3 4 5 6 7 8 9 10 11 12 13 14 15 16 17 18 19 20 21 22 23 24 25 26 27 28 29 30 31 32 33 34 35 36 37 38 39 40 41 42 43 44 45 46 47
Sampling sites

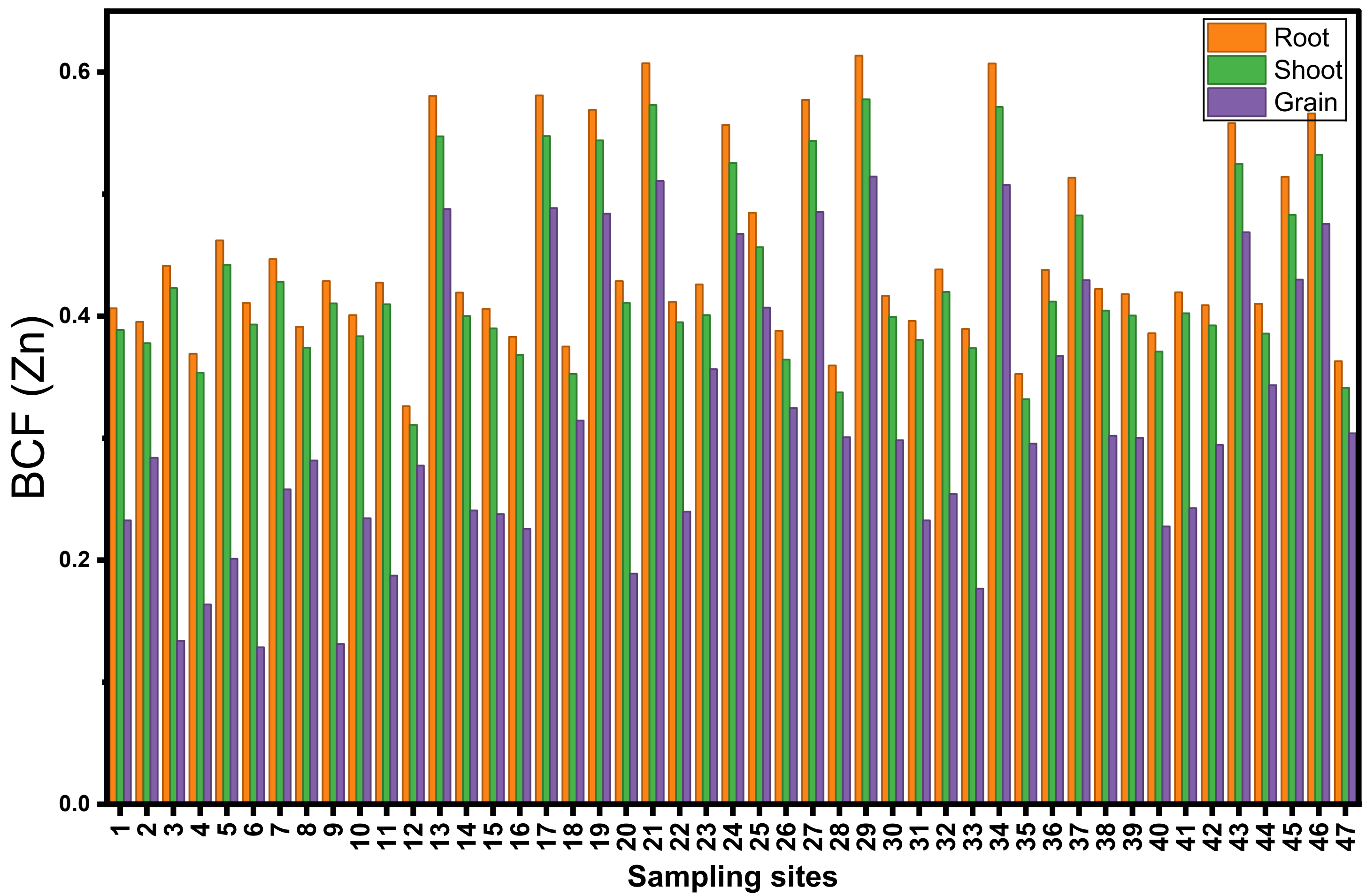

Root
Shoot
Grain
BCF (Zn)
0.0
0.2
0.4
0.6
Sampling sites
1 2 3 4 5 6 7 8 9 10 11 12 13 14 15 16 17 18 19 20 21 22 23 24 25 26 27 28 29 30 31 32 33 34 35 36 37 38 39 40 41 42 43 44 45 46 47

Fig. S1. Figure 6**.** Bioconcentration factor (BCF) showing variation in metal accumulation among different parts of the rice plant (root, shoot, and grain) across the sampling sites.

References

Chen, R.H., Chen, H.Y., Song, L.T., Yao, Z.P., Meng, F.S., Teng, Y.G., 2019. Characterization and source apportionment of heavy metals in the sediments of Lake Tai (China) and its surrounding soils. Sci Total Environ 694.

Gong, C., Quan, L., Chen, W., Tian, G., Zhang, W., Xiao, F. and Zhang, Z., 2024. Ecological risk and spatial distribution, sources of heavy metals in typical purple soils, southwest China.Scientific Reports,14(1), p.11342.

Han, Q., Wang, M., Cao, J., Gui, C., Liu, Y., He, X., He, Y., Liu, Y., 2020. Health risk assessment and bioaccessibilities of heavy metals for children in soil and dust from urban parks and schools of Jiaozuo, China. Ecotoxicol Environ Saf 191, 110157.

Huang, J.H., Guo, S.T., Zeng, G.M., Li, F., Gu, Y.L., Shi, Y.H., Shi, L.X., Liu, W.C., Peng, S.Y., 2018. A new exploration of health risk assessment quantification from sources of soil heavy metals under different land use. Environ Pollut 243, 49-58.

Hakanson, L. (1980). An ecological risk index for aquatic pollution control.a sedimentological approach. Water Research, 14(8), 975–1001.

Huang, J.L., Wu, Y.Y., Sun, J.X., Li, X., Geng, X.L., Zhao, M.L., Sun, T., Fan, Z.Q., 2021. Health risk assessment of heavy metal(loid)s in park soils of the largest megacity in China by using Monte Carlo simulation coupled with Positive matrix factorization model. Journal of Hazardous Materials 415, 11.

Huang, J.H., Guo, S.T., Zeng, G.M., Li, F., Gu, Y.L., Shi, Y.H., et al., 2018. A new exploration of health risk assessment quantification from sources of soil heavy metals under different land use. Environ. Pollut. 243, 49–58.

Müller, G., 1969. Index of geoaccumulation in sediments of the Rhine Rive. Geojournal 2, 108-118.

Tomlinson, D. L., Wilson, J. G., Harris, C. R., & Jeffrey, D. W. (1980). Problems in the assessment of heavy-metal levels in estuaries and the formation of a pollution index. Helgoländer Meeresuntersuchungen, 33(1–4), 566–575.

USEPA, 1992. Guidelines for exposure assessment. *Federal Register*, *57*(104), pp.22888-938.

Wang, G., Liu, H.Q., Gong, Y., Wei, Y., Miao, A.J., Yang, L.Y., Zhong, H., 2017. Risk assessment of metals in urban soils from a typical industrial city, Suzhou, Eastern China. Int J Environ Res Public Health 14.

Wang, X. F., Deng, C. B., Sunahara, G., Yin, J., Xu, G. P., Zhu, K. X., 2020. Risk assessments of heavy metals to children following non-dietary exposures and sugarcane consumption in a rural area in Southern China. Exposure and Health, 12(1), 1-8. doi.org/10.1007/s12403-018-0275-0

Yang, S.Y., Zhao, J., Chang, S.X., Collins, C., Xu, J.M., Liu, X.M., 2019. Status assessment and probabilistic health risk modeling of metals accumulation in agriculture soils across China: A synthesis. Environ Int 128, 165-174.

Zhou, Y., Jiang, D.D., Ding, D., Wu, Y.J., Wei, J., Kong, L.Y., Long, T., Fan, T.T., Deng, S.P., 2022. Ecological-health risks assessment and source apportionment of heavy metals in agricultural soils around a super-sized lead-zinc smelter with a long production history, in China. Environmental Pollution 307, 11.
